# Rationally designed minimized TbpB confers broad protection against meningococcal infection

**DOI:** 10.1371/journal.ppat.1014493

**Published:** 2026-08-10

**Authors:** Epshita A. Islam, Jamie E. Fegan, Gregory B. Cole, Charles Calmettes, Dixon Ng, Natalie Y. T. Au, Carolyn Marion, Sang K. Ahn, David M. Curran, Laura-lee Caruso, Anthony B. Schryvers, Trevor F. Moraes, Scott D. Gray-Owen

**Affiliations:** 1 Department of Biochemistry, Temerty Faculty of Medicine, University of Toronto, Toronto, Canada; 2 Department of Molecular Genetics, Temerty Faculty of Medicine, University of Toronto, Toronto, Canada; 3 Department of Microbiology, Immunology, and Infectious Diseases, Cumming School of Medicine, University of Calgary, Calgary, Canada; Institut de Microbiologie de la Mediterranee, FRANCE

## Abstract

Transferrin binding protein B (TbpB), an iron acquisition protein, has long been recognized as a promising vaccine candidate targeting the pathogenic Neisseria species, including *Neisseria meningitidis*, the cause of meningococcal disease, and *Neisseria gonorrhoeae*, the cause of gonorrhea. A challenge to the development of this protein as a vaccine immunogen is the extent of antigenic variability it exhibits, which complicates the selection of a single variant to elicit a broadly cross-protective immune response. We have utilized structure-informed antigen engineering to develop a minimized version of TbpB consisting of the protein’s carboxy-terminal lobe with its variable surface loops removed. Here, we reveal the effectiveness of this “loopless C-lobe” as an independent immunogen, with structural characterization and stability studies to demonstrate its integrity, and murine immunization and challenge studies that establish its ability to elicit robust protective efficacy by using models of *N. meningitidis* invasive infection and nasopharyngeal colonization. The breadth of protection provided, as measured by both in vitro analysis and cross-protection mouse challenge studies, indicate that a single loopless C-lobe elicits a broadly cross-protective immune response against the diverse panel of meningococcal strains tested, and that the cross-reactivity is superior to that offered by the intact TbpB or the native C-lobe. Together, this study demonstrates the utility of structure-informed antigen engineering towards the development of broadly efficacious protein-based vaccines.

## Author summary

Surface-exposed proteins on bacterial pathogens are enticing candidate vaccine targets, however their exposure to the immune system frequently leads to high levels of antigenic variation, a factor that complicates the development of broadly protective vaccines. Here, we undertake an antigen engineering approach to develop a minimized version of a surface lipoprotein, transferrin binding protein B, where variable regions of the protein have been removed to focus the immune response to conserved regions of this antigen. We combine structural studies and mouse infection models of *Neisseria meningitidis*, the cause of meningococcal disease, and *Neisseria gonorrhoeae*, the causative agent of gonorrhea, to evaluate the effect that our strategic minimizing of the protein immunogen has and demonstrate it alters the immune response against TbpB to extend the resulting breadth of cross-reactivity and cross-protection against diverse meningococcal isolates.

## Introduction

Surface lipoproteins (SLPs) have long been viewed as promising vaccine targets for gram-negative bacterial pathogens, including the pathogenic Neisseria species, due to their surface accessibility, importance in pathogenesis, and inherent stability as potential immunogens [1–6]. SLPs typically play crucial roles in host-pathogen interactions, including nutrient acquisition, immune evasion, and cell adhesion, indicating that these targets are unlikely to be lost due to selective pressures [7]. However, the surface accessibility required to facilitate binding to host ligands inevitably exposes SLPs to the host immune system, often leading to substantial antigenic variation of these proteins [8,9]. This poses a major challenge for developing SLP-based vaccines with broad coverage.

Despite the inherent variability of these antigens, SLPs have been successfully used in commercial vaccines. Notably, factor H binding protein (fHbp), which helps *Neisseria meningitidis* (Nme) evade the host complement response by binding the inhibitory protein Factor H, is found in two separate commercially licensed protein-based serogroup B meningococcal (MenB) vaccines. To counteract the antigenic variation of this protein, the bivalent MenB vaccine (Trumenba, Pfizer) uses two fHbp variants while the 4CMenB vaccine (Bexsero, GSK) is composed of a single fHbp antigen along with three additional bacterial components to expand coverage [9,10].

The bipartite bacterial transferrin receptor, which is composed of transferrin binding protein B (TbpB, an anchored SLP) and transferrin binding protein A (TbpA, an integral outer membrane protein), is a highly promising vaccine target with well-established roles in invasive disease and mucosal persistence for a variety of human and animal pathogens from the *Neisseriaceae*, *Pasteurellaceae,* and *Moraxellaceae* families [11–14]. This receptor functions for bacterial iron acquisition, where the surface-anchored TbpB extends away from the bacterial surface and binds to iron-loaded (holo) host transferrin (Tf) [15], followed by removal and internalization of iron across the outer membrane through TbpA [16]. Several studies by our group and others have demonstrated the efficacy of recombinant, purified TbpB or TbpA proteins in mouse immunization and challenge studies against Nme, the causative agent of invasive meningococcal disease and *Neisseria gonorrhoeae* (Ngo), the causative agent of gonorrhea. The benefit of targeting these antigens lies in their ability to not only prevent invasive disease, but also potentially extend protection to relevant mucosal surfaces [5,6,17]. This latter effect is an essential requirement for any gonococcal vaccine in development and would be a significant improvement over the current Group B meningococcal vaccines that have limited impact on nasopharyngeal carriage [18–20].

Vaccine efforts have largely focused on TbpB due to its inherent stability, ease of production, and scalability for translation applications. Neisserial TbpBs segregate into distinct lineages, Isotype I and Isotype II, with minimal cross-reactivity or protection being elicited between the two isotypes [21]. The Isotype I lineage is comprised solely of meningococcal TbpBs, while Isotype II can be divided into four meningococcal and two gonococcal sub-clusters according to our most recent phylogenetic analysis [22]. TbpB consists of two lobes, C-lobe and N-lobe, with the latter binding to the C-lobe of holo human transferrin (hTf) [23]. The sequence variability in TbpB across strains mainly resides within the more distal, transferrin-binding interface of the N-lobe, with the β-barrel core of the C-lobe being relatively conserved [8,24]. We have recently demonstrated that a composition consisting of two rationally selected full-length Ngo TbpBs is sufficient for broad Ngo coverage [22]. As the meningococcal TbpBs are more diverse, we postulate that multiple Isotype II variants in combination with an Isotype I TbpB may be necessary for broad meningococcal coverage if a vaccine were to consist of only full-length TbpB antigens. While feasible, multi-antigen vaccines require greater production requirements and result in more expensive end products, thereby limiting accessibility.

Towards achieving effective coverage with a minimum number of antigens, our group has engineered a minimized version of an Isotype II Nme TbpB C-lobe, termed the Loopless C-Lobe (LCL). The LCL was generated by exploiting the solved crystal structure of serogroup B strain M982 by deleting the N-lobe sequence and replacing four relatively large, variable loops (26, 30, 21, and 19 amino acids in length) within the C-lobe that were not resolved in the crystal structure [24] with smaller fragments (2–6 amino acids) from loop regions of *Actinobacillus pleuropneumoniae* TbpB [25]*.* This variant was chosen as Isotype II represents the majority of meningococcal TbpBs and the solved crystal structure allowed structure-informed antigen engineering to be performed. We postulated that the LCL could serve as an antigen with dual modality. First, being an easily scalable, soluble protein, LCL could be utilized as a scaffold to display surface-exposed loops from integral membrane proteins that are otherwise challenging to produce at large scale. Second, by removing the more hypervariable N-lobe and the C-lobe loops, the remaining LCL sequence is highly conserved across the Nme and Ngo TbpB proteome. Therefore, we considered whether immunization with LCL may elicit immune responses to conserved portions of diverse TbpBs, thereby improving coverage.

In support of its utility as a scaffold, we have previously demonstrated that LCL-TbpA hybrids, wherein different surface exposed loops from TbpA were grafted on to LCL, can elicit TbpA-specific antibodies with bactericidal activity and immunization with these immunogens elicited protective immunity in mouse challenge models [17]. We have also shown that LCL-based hybrids displaying loops of the zinc-binding protein ZnuD from *Acinetobacter baumannii* can protect against this pathogen in a mouse sepsis model [26], and the LCL has also been used to display extracellular loops from outer membrane proteins from *Treponema pallidum* [27]. Here we establish the protective capacity of LCL as a stand-alone antigen, demonstrating the extent of anti-LCL antibody coverage of heterologous TbpBs and the efficacy of LCL against heterologous *N. meningitidis* and *N. gonorrhoeae* strains in mouse sepsis and colonization models.

## Results

### Structure and stability of LCL

**Fig 1:**
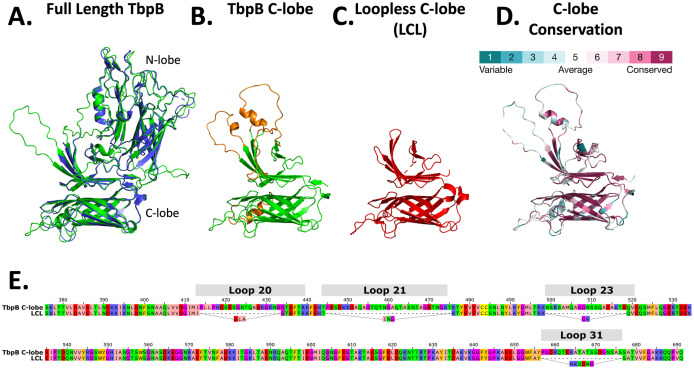
Structural analysis of TbpB and its variants. A: Full-length M982 TbpB structure, solved by X-ray crystallography (PDB 3VE2) [24], is aligned to the full-length structure predicted by AlphaFold 3 [28]. Several large loops are not observed in the crystal structure and are predicted to be disordered. B: The AlphaFold 3 predicted structure of M982 TbpB C-lobe; loops that we replaced with shorter heterologous loops in the LCL are shown in orange. C: Structure of the LCL (PDB 5KKX) solved by X-ray crystallography. D: Sequence conservation in the C-lobe calculated using our previous phylogenetic analysis [22] and the ConSurf web server [29]. E: Amino acid sequence alignment of the TbpB C-lobe of M982 with the engineered LCL. Loops selected for removal are indicated, with residues used to ‘cap’ the removed loops shown in the center of the loop gap in the LCL.

To create the LCL, we replaced the four native C-lobe loops of M982 TbpB that were not resolved in the full-length crystal structure (PDB 3VE2) [24] with heterologous linkers. AlphaFold models [28] predict that the deleted loop regions are largely unstructured (Fig 1A, 1B). To determine if loop replacements had an impact on the overall structure of the C-lobe core, we solved the crystal structure of LCL (PDB 5KKX) (Fig 1C), which demonstrated that the core structure remains intact. To visualize the conservation of amino acids within the C-lobe, we used the Consurf webserver and an alignment of 1,485 publicly available TbpB variants. Each amino acid position was assigned a class (1 being the most variable through 9 representing the most conserved) and was projected onto the C-lobe structure, where highly variable regions are coloured turquoise and highly conserved regions are coloured maroon. Notably, these conservation scores are relative conservation measurements calculated by the Rate4Site algorithm and do not necessarily equate to strict conservation percentages. Overall, this analysis shows that the LCL portion is highly conserved, while the four deleted loops contained more variability (Fig 1D). A sequence alignment of the C-lobe of TbpB against the LCL shows the locations of the four deleted loops (Fig 1E).

**Fig 2:**
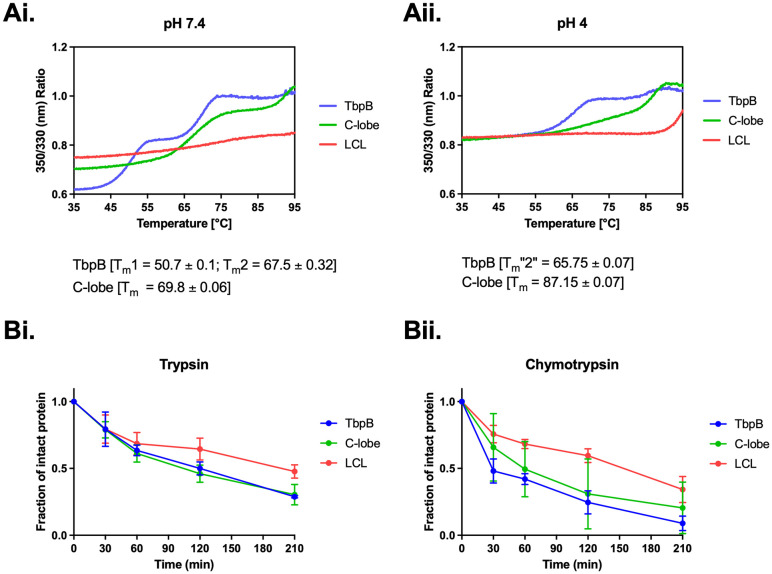
Comparing thermal stability and proteolysis of intact TbpB, C-lobe, and LCL. Ai, Aii: Thermal stability of TbpB, C-lobe and LCL at neutral and acidic pH, respectively, measured using NanoTemper Tycho with the Tm values indicated below. Bi, Bii: Graphs depicting the fraction of intact protein over time after treatment with trypsin and chymotrypsin, respectively. For trypsin, mean + /- standard deviation shown; n = 3 technical replicates per group per time point. For chymotrypsin, mean + /- standard deviation shown; n = 4 technical replicates (TbpB and LCL) or n = 2 (C-lobe) per time point.

We next assessed the thermal stability of each recombinant protein (full-length TbpB, C-lobe, LCL) by measuring the intrinsic fluorescence signal from tryptophan and tyrosine residues using nano differential calorimetry (nanoDSF) [30], at neutral and acidic pHs to mimic conditions in the extracellular space and the endosome, respectively (Fig 2A). Full-length TbpB displayed two transition temperatures (~50^o^C and ~70^o^C), which we presume correspond to the unfolding of the N- and C-lobes, as the C-lobe had a single transition (at ~70^o^C) that closely matched the second transition of full-length TbpB. Notably, the LCL fluorescence emission ratio at 350nm/330nm increased slightly but did not produce an inflection point, likely due to the β-barrel remaining mostly intact even at elevated temperatures. To further examine the stability of the LCL, proteolytic cleavage by trypsin and chymotrypsin was performed and monitored over time. LCL displayed greater resistance to both enzymes compared to its parental full-length TbpB and C-lobe constructs (Fig 2B, representative original gel images are available as S1 Fig). Overall, these studies confirm that LCL is a highly stable antigen that closely recapitulates the core tertiary structure of the TbpB C-lobe.

### Immunization with LCL protects against meningococcal sepsis by the homologous meningococcal strain

**Fig 3:**
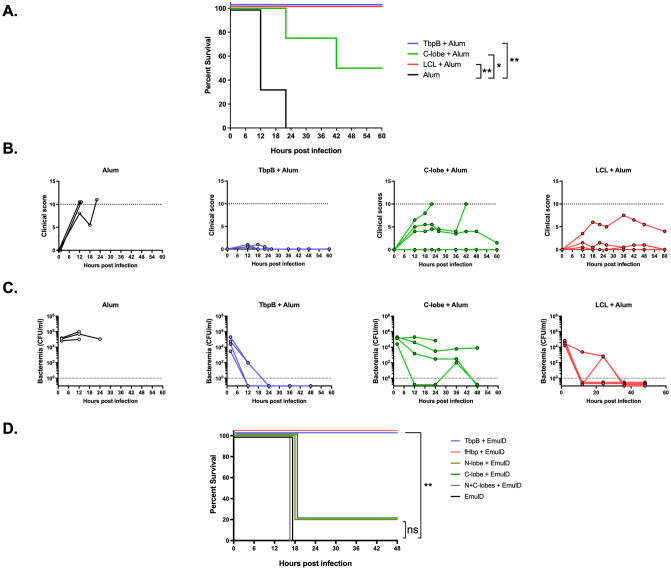
Comparison of TbpB, C-lobe, and LCL to elicit protection against meningococcal sepsis. A: Percentage of survivors after intraperitoneal administration of a lethal dose of the homologous M982 Nme strain. N = 3-4 mice per group. B: Cumulative clinical score of individual animals with the group indicated above each graph. Dotted line at 10 depicts the clinical score cutoff for humane endpoint. C: Bacterial recovery from tail vein bleeds from the time points indicated. Dotted line at 1 depicts the detection limit. D: Percentage of survivors after intraperitoneal administration of a lethal dose of the homologous M982 Nme strain in mice immunized with full length TbpB, full length fHbp, individual lobes of TbpB, or individual lobes of TbpB mixed together, each formulated with the oil-in-water adjuvant Emulsigen-D. N = 5 mice per group. p-value of survival curves calculated using Log-rank (Mantel-Cox) test comparing each immunized group against mice that received adjuvant only; *, p < 0.05; **, p < 0.01. Statistical analysis performed using GraphPad Prism 10.2.0.

To compare the protection elicited by LCL to its parental TbpB and full-length C-lobe, C57BL/6 male mice were immunized three times and then challenged with a lethal dose of the homologous *N. meningitidis* M982 strain in a sepsis challenge model (Fig 3). As expected, mice that received adjuvant only reached clinical endpoint by 24 hours post infection (0/3 survivors) and were highly bacteremic. All animals immunized with full-length M982 TbpB survived the challenge (4/4 survivors), developed minimal symptoms, and cleared bacteria from the blood by 24 hours post infection. M982 C-lobe immunized group had 50% protection (2/4 survivors), with both survivors clearing bacteria by 48 hours. The loss of protection when using individual lobes of TbpB has been a consistent trend in both *N. meningitidis* mouse sepsis studies (Fig 3D) and in pigs immunized with full-length TbpB or individual lobes derived from the porcine pathogen *Glaesserella parasuis* [31]. Unexpectedly, this loss of protection was maintained when the two individual lobes were mixed together and used in a vaccine formulation, indicating that epitopes spanning both lobes when maintained in a single antigen may be integral for eliciting a protective immune response when targeting full length TbpB.

Strikingly, mice that received LCL were fully protected from this challenge (4/4 survivors). Clinical scores showed 3 out of 4 mice were protected from clinical symptoms, with the one symptomatic mouse recovering by the end of the study. 2 out of 4 mice cleared bacteria within 12 hours and the remaining animals cleared by 36 hours post infection. These results demonstrate that LCL is a superior antigen to the C-lobe and can yield comparable protection to full-length TbpB against the homologous strain during invasive challenge.

### LCL protects against nasopharyngeal colonization by the homologous meningococcal strain

Next, we utilized human carcinoembryonic antigen-related cell adhesion molecule 1 (hCEACAM1)- expressing transgenic mice which are permissive to Nme colonization of the nasopharynx after direct nasal instillation [32]. Using this model, we have previously reported that the 4CMenB vaccine can reduce nasopharyngeal carriage of only a subset of singleantigen matched meningococcal strains, suggesting that not all antigens included in the 4CMenB formulation are able to provide adequate mucosal protection after parenteral vaccination [20]. In direct support of this premise, comparison of fHbp to full-length TbpB revealed that while both fully protect against sepsis and elicit significant bactericidal titres (Figs 3D, S2B), only full-length TbpB reduces nasal colonization rates (from 64% for adjuvant and 58% for fHbp groups to 18% for the TbpB-immunized group, S2A Fig). Therefore, we proceeded to evaluate if LCL can retain the protection elicited by full-length TbpB.

**Fig 4:**
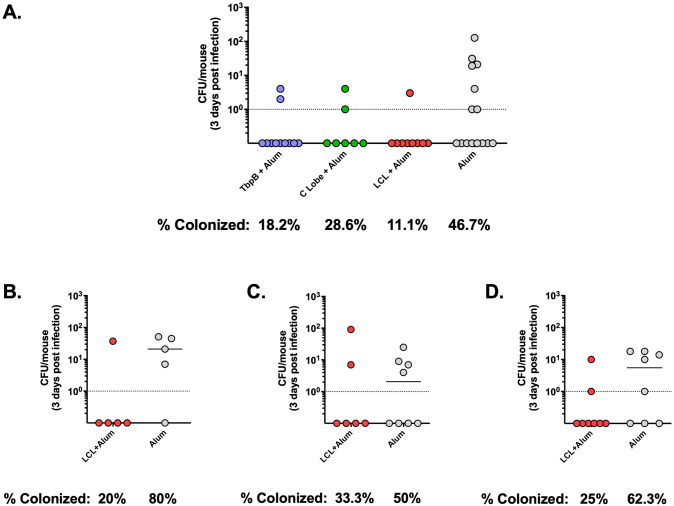
LCL-mediated reduction in nasal colonization by the homologous Nme M982 strain. Meningococcal recovery from the nasopharynx of immunized hCEACAM1 transgenic mice 3 days post nasal administration of the homologous M982 Nme strain, with the percentage of culture positive animals indicated below. A: Comparing protection mediated by TbpB, C-lobe, and LCL intraperitoneal immunization in hCEACAM1 + /- (mCEACAM1 + /+) FvB mice. N = 7-15 for each group. B-D: Comparing LCL to adjuvant control in 3 additional independent experiments with the following conditions: B: intraperitoneally immunized hCEACAM1 + /- (mCEACAM1 + /+) FvB mice; C: subcutaneously immunized hCEACAM1 + /- (mCEACAM1-/-) C57BL/6 mice; D: intraperitoneally immunized hCEACAM1 + /- (mCEACAM1-/-) C57BL/6 mice. N = 5-8 for each group. Each circle represents Nme recovered from one mouse, line at median. One-way ANOVA with Dunnett’s multiple comparison of the bacterial burden in each vaccinated group to the control (A) and non-parametric Mann-Whitney test (B-D) performed using GraphPad Prism 10.2.0 did not yield significant p-values.

Full-length TbpB, C-lobe, and LCL-immunized hCEACAM1 FvB mice were intranasally challenged with the homologous Nme M982 strain and the bacteria recovered from the nasopharyngeal tissue was enumerated three days post infection. The percentage of culture positive animals in the adjuvant control group was 46.7% (7/15 mice), while full-length TbpB, C-lobe, and LCL-immunized groups had reduced colonization rates of 18.2% (2/11), 28.6% (2/7), and 11.1% (1/9) respectively (Fig 4A). Since M982 is not a robust colonizer of the murine nasopharynx and to ensure reproducibility, we performed three additional independent studies comparing only LCL to adjuvant in hCEACAM1 transgenic mice from different genetic backgrounds and varying routes of immunization. In each of these studies, the colonization rate in LCL-immunized animals were consistently lower than adjuvant controls: LCL+Alum – 20% (1/5), 33% (2/6), 25% (2/8) versus Alum – 80% (4/5), 50% (4/8), 62.5% (5/8) respectively, although the reduction in bacterial burden did not reach statistical significance (Fig 4B, 4C, 4D). These results demonstrate that parenteral immunization with LCL provides consistent reduction in nasal colonization rates, with its performance being comparable to full-length TbpB against the homologous challenge strain.

### LCL elicits functional IgG against TbpB

**Fig 5:**
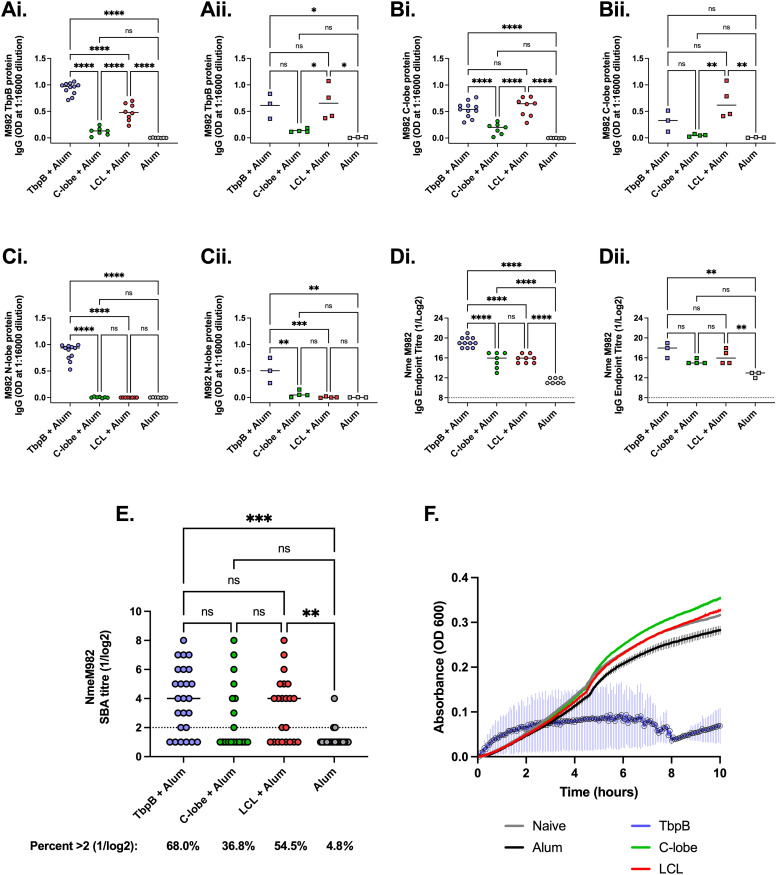
Comparison of anti-TbpB antibodies elicited by TbpB, C-Lobe, and LCL antigens. A-C: Protein ELISA measuring reactivity of TbpB, C-lobe, and LCL-immune sera against full-length TbpB, TbpB C-lobe, and TbpB N-lobe, respectively. D: IgG titre elicited by the different immunogens towards heat inactivated Nme M982. Panel i (circles) represent terminal serum from hCEACAM1 FvB mice from the colonization study presented in Fig 3A, N = 7-11 mice per group; panel ii (squares) represent pre-challenge sera from C57BL/6 animals from the sepsis study presented in Fig 2, N = 3-4 mice per group; each symbol represents serum from one animal tested in duplicate. E: Serum bactericidal activity (SBA) titre against iron-starved Nme M982. N = 19-25 per group, including terminal hCEACAM1 FvB serum from Fig 4A, plus additional terminal serum from immunized hCEACAM1 FvB from pilot studies. Bactericidal titre (>50% killing at a 1:8 serum dilution minimum) reached for 17/25 (68.0%) TbpB, 7/19 (36.8%) C-lobe, 12/22 (54.5%) LCL and 1/21 (4.8%) Alum samples. Dotted lines represent lowest dilution tested. Line at median for each group. One-way ANOVA with Tukey’s post-hoc test comparing each group to every other group was performed using GraphPad Prism 10.2.0. For D, E: Statistics performed on Log2 transformed data. ns, not significant; **, p < 0.01; ***, p < 0.001; ****, p < 0.0001. F: Meningococcal growth inhibition assay. Iron-starved Nme M982 was grown in the presence of hTf and pooled heat-inactivated serum from TbpB, C-lobe and LCL immunized animals from Fig 4A. Change in absorbance (OD600) relative to the initial time point is graphed; error bars depict standard deviation of two technical replicates.

Protection against invasive meningococcal infection is antibody-dependent [33]. Considering that LCL and full-length TbpB, but not C-lobe, fully protected in the sepsis model, we measured anti-TbpB serum IgG levels among these groups. Pre-challenge serum from the sepsis study in Fig 2 and terminal serum from the colonization study in Fig 3A were assayed using protein-based and heat-inactivated whole bacterial ELISAs (Fig 5A-5C, 5D respectively). Of note, terminal serum is not reliably collected after invasive challenge due to high levels of dehydration in animals with substantial clinical symptoms, and biosafety concerns in handling serum from mice with *N. meningitidis* bacteremia, however larger volumes of serum available from terminal blood collection from mice challenged on a mucosal surface expands the scope of serology that can be performed. As expected, immunization with full-length TbpB resulted in the highest antibody titre against the full protein and whole bacteria, with recognition of both C- and N-lobes, whereas immunization with the C-lobe resulted in significantly lower anti-TbpB antibodies and no N-lobe reactivity. Compared to C-lobe, immunization with LCL yielded significantly higher IgG serum reactivity at the dilution tested in protein-based ELISAs (Fig 5A, 5B), implying that the latter is more immunogenic. Regardless of antigen, these TbpB-based formulations elicited anti-TbpB IgG1, IgG2a, and IgG2b, however limited serum IgG3, IgA, or IgM was elicited (S3A-G Fig). Anti-TbpB IgG was also detected in nasal lavage samples, however neither mucosal IgA or IgM was detected after immunization (S3H Fig). In contrast, the difference in serum reactivity against whole bacteria between LCL and C-lobe sera was subtle (Fig 5D), presumably due to proximity of other membrane proteins and/or the polysaccharide capsule limiting antibody access, or artifacts arising during plate preparation masking some epitopes.

Serum bactericidal activity has long served as a reliable correlate of protection against invasive meningococcal disease and is considered the gold standard in the field [33]. Bactericidal titre also correlated with protection in our comparative study of fHbp and TbpB (S2B Fig, where only the groups that were protected in the sepsis study in Fig 3D had significant bactericidal titres). Therefore, to elucidate antibody-dependent mechanisms contributing to LCL-mediated protection, we next performed serum bactericidal assays. Both anti-LCL and anti-TbpB serum had significant bactericidal activity, while anti-C-lobe serum had lower bactericidal titres (Fig 5E), reflecting the protection outcome observed in the sepsis challenge. Of note, while we have not seen infection-based boosting of anti-TbpB antibody titres, serum used here was post immunization and post infection, thus we cannot rule out an increase in bactericidal titres based on exposure to the pathogen during infection. We additionally compared the ability of these sera to directly block hTf-dependent bacterial growth. With full-length TbpB antigens, we consider this additional mechanism of nutritional deprivation by antibodies competing for the transferrin binding site on TbpB important for mediating protection [34]. Although LCL antibodies do not bind the N-lobe of TbpB where the transferrin-binding interface lies, we wondered whether antibodies binding to the C-lobe could affect transferrin utilization indirectly by hindering either TbpB-hTf and/or TbpB-TbpA interactions. To test this, we compared the growth of M982 Nme in the presence of serum and hTf. Only TbpB serum, but not LCL or C-lobe serum, was able to inhibit transferrin dependent bacterial growth (Fig 5F). Taken together, these results suggest that LCL antibodies facilitate bacterial clearance through complement-mediated bacterial lysis, but not nutrient starvation.

### Anti-LCL antibodies are cross-reactive against diverse meningococcal TbpBs

**Fig 6:**
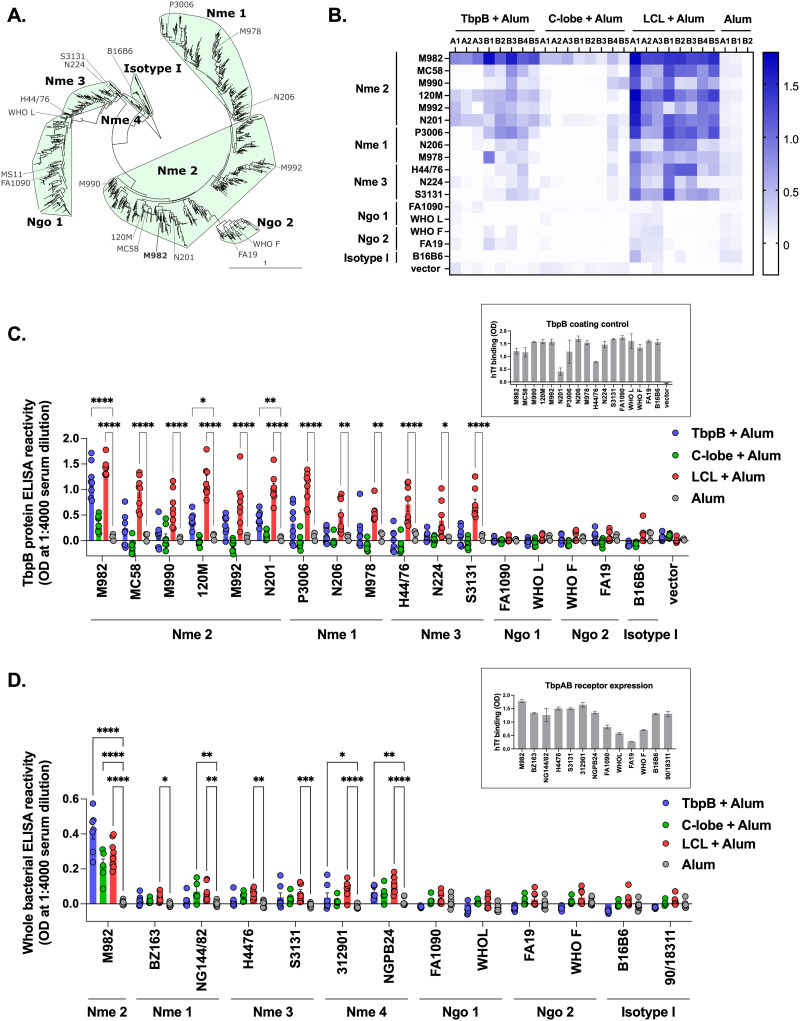
Antibody cross-reactivity against TbpB proteins. A: Phylogenetic tree depicting Neisserial TbpB diversity. The location of M982 TbpB is highlighted, along with 16 additional TbpBs spanning various clusters used for cross-reactivity analysis. Phylogenetic tree adapted from Fegan *et al,* 2025 [22]. B: Heat map depicting IgG cross-reactivity against a broad panel of TbpB protein variants by ELISA. Optical density (OD) readings denoted by the colour gradient. Background noise from no-protein control has been subtracted. Each row is a different TbpB protein capture, with the phylogenetic cluster indicated to the left. Each column is a different mouse sample, with the immunizing antigen indicated above. Numbered labels starting with A are pre-challenge serum from the immunized C67BL/6 sepsis cohort; B are terminal serum from the immunized hCEACAM1 FvB colonization cohort. C: Grouped bar graph summarizing cross-reactive antibody data from B. Bars represent mean, error bars represent standard deviation, each circle represents serum from a single mouse (N = 8 for immune sera, N = 3 for Alum). hTf-HRP signal quantifying properly folded TbpB capture is depicted in the inset, mean ± standard deviation. D: Serum reactivity against representative Nme and Ngo strains grown under iron limitation using inactivated whole bacterial ELISA. TbpB cluster indicated below the x-axis. Background noise from no serum control has been subtracted. Bars represent mean, error bars represent standard deviation, each circle represents serum from a single mouse, N = 5-8/group. Inset depicts hTf-binding as an indicator of TbpAB receptor expression on bacteria used for coating ELISA plates, mean ± standard deviation. For C and D, two-way ANOVA with Dunnett’s post-hoc test comparing each group to Alum control performed using GraphPad Prism 10.2.0. Only p-values <0.05 shown. *, p < 0.05; **, p < 0.01; ***, p < 0.001, ****, p < 0.0001.

Since the LCL was engineered to remove most of the hypervariable sequences within TbpB, we next examined whether this strategy results in broadly cross-reactive antibodies being raised against conserved regions of TbpB. Phylogenetic analysis of all publicly accessible TbpB protein sequences from Nme and Ngo results in the sequences dividing into several clusters, with the meningococcal Isotype I TbpBs forming a single cluster (Isotype I) and Isotype II TbpBs separating into four Nme (Nme 1–4) and two Ngo clusters (Ngo 1, 2) (Fig 6A, sequence alignment of the TbpB C-lobes of included variants is available in S4 Fig). To evaluate cross-reactivity of immune sera, we first selected representative TbpBs from different clusters (N = 3 from Nme 1; N = 6 from Nme 2, including the homologous M982 TbpB; N = 3 from Nme 3; N = 2 from Ngo 1; N = 2 from Ngo 2, N = 1 from Isotype I) for high throughput protein ELISAs [35]. Signal obtained against each TbpB is depicted as a heat map to showcase individual responses and as bar graphs for overall group trends (Fig 6B and 6C, respectively). Signal from HRP-labelled hTf (hTf-HRP) were used to confirm uniform coating of the ELISA plate with properly folded analyte (Fig 6C, inset).

Despite high reactivity against the homologous TbpB, serum from full-length TbpB immunized animals reacted weakly against heterologous TbpBs. Reactivity varied based on mouse background, with C57BL/6 serum only recognizing a subset of Nme 2 TbpBs and hCEACAM1 FvB serum recognizing additional Nme 1 and Nme 3 TbpBs (Fig 6B). Overall, significant cross-reactivity with anti-TbpB IgG was only observed against two Nme 2 TbpBs: TbpBs from serogroup A strains 120M and N201 (Fig 6C). C-lobe sera reacted primarily to the homologous M982 TbpB, with only a single hCEACAM1 FvB sample producing a broader reactivity pattern. Remarkably, serum from LCL-immunized animals recognized all Nme Isotype II TbpBs in our panel. This LCL-elicited response was significant and consistent regardless of mouse background.

Next, we examined cross-reactivity against a panel of Nme and Ngo strains from all heterologous TbpB clusters (N = 2 strains from Nme 1, 3, 4, Ngo 1, 2, Isotype I; N = 5–8 for each serum group) (Fig 6D; inset depicts hTf-binding as an indicator of transferrin receptor surface expression, however this control cannot discern between TbpA and TbpB binding). Individual mouse serum was tested at a 1:4,000 dilution as this dilution consistently shows robust signal in whole cell ELISAs when testing immune sera against the strain expressing the homologous TbpB. Consistent with the pattern observed with protein-based ELISAs, anti-LCL antibodies significantly cross-reacted against all Nme Isotype II strains, increasing strain coverage compared to full-length TbpB antigen which only had significant cross-reactivity signal against strains from the Nme 4 cluster. We further performed western blots on the same strain panel to confirm that LCL serum is specifically recognizing TbpBs (S5 Fig) and confirmed growth on deferoxamine mesylate salt (‘Iron Starved’ condition) led to expression of TbpB by both antibody recognition and human transferrin binding compared to growth on rich media (S6 Fig). To confirm the reactivity seen against heterologous strains, additional dilutions of pooled serum were evaluated against eight of the meningococcal strains of interest to confirm the reactivity panel shown in Fig 6D (S7 Fig).

While characterization of these cross-reactive antibodies is beyond the scope of this study, the consistent recognition of Isotype II Nme TbpBs, but not Ngo TbpBs, leads to the consideration of whether there are regions within the LCL that are conserved in Nme TbpBs, and distinct in Ngo TbpBs, that could provide clues regarding the location of potential cross-reactive epitopes.

### LCL immunization improves clinical outcome during invasive infection by heterologous Isotype II Nme strains

**Fig 7:**
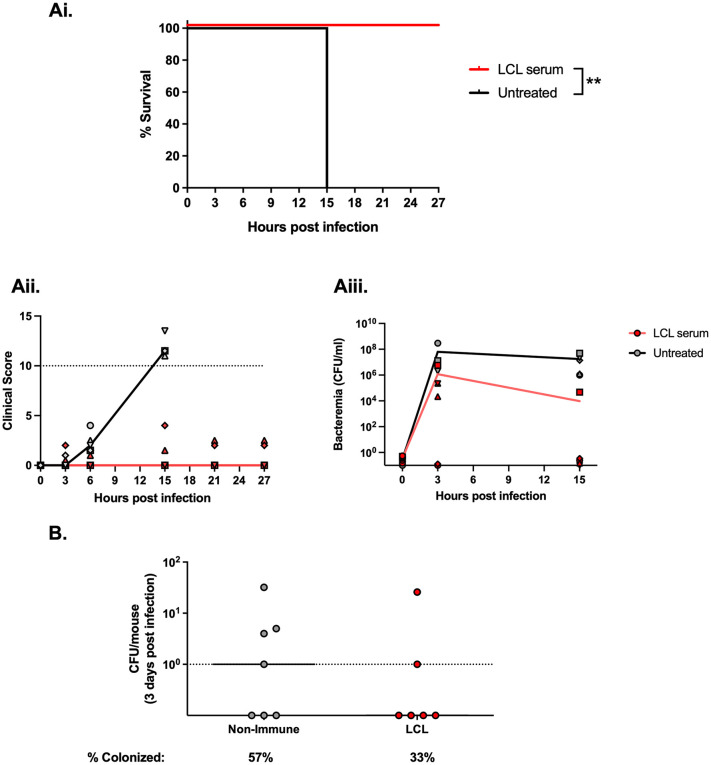
Passive immunization with LCL serum protects against invasive challenge. Ai: Survival, Aii: clinical scores, and Aiii: bacteremia after intraperitoneal injection with Nme M982 in C57BL/6 mice that were untreated versus treated with inactivated rabbit LCL antiserum 6 hours prior to infection. N = 5 mice per group; p-value calculated using Log-rank (Mantel-Cox) test; **, p < 0.01. Dotted line at 10 depicts in the clinical score graph represents cutoff for humane endpoint. B: Meningococcal burden in the nasopharynx of hCEACAM1 + /- (mCEACAM1 + /+) FvB mice that were either untreated or intraperitoneally injected with LCL serum 20 hours prior to nasal infection with the homologous M982 Nme strain, with the percentage of culture positive animals indicated below. Line depicts the limit of detection.

We performed passive transfer studies using anti-LCL immune serum in mice followed by either invasive challenge (Fig 7A) or nasal infection (Fig 7B) and demonstrated reduced infection in mice that received anti-LCL sera compared to control animals in each model, providing direct evidence of antibody-mediated protection in these infection models. This, combined with the broad strain recognition by anti-LCL antibodies (Fig 5) prompted us to conduct challenge studies with strains from each heterologous Nme Isotype II (Nme 1, 3, 4) and Isotype I clusters to assess the breadth of cross protection.

**Fig 8:**
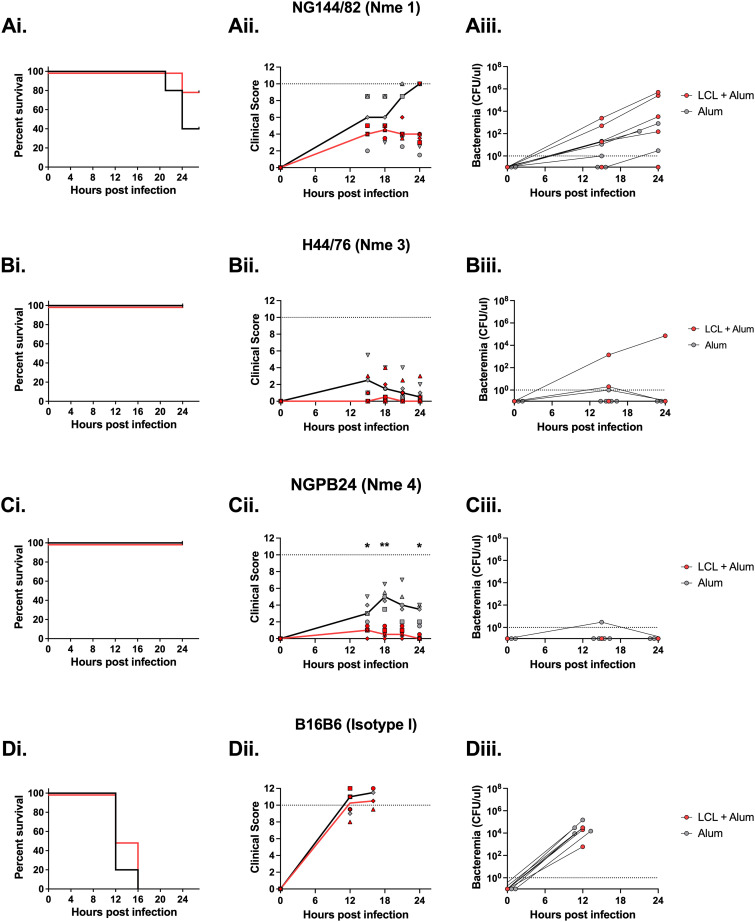
Reduction in severity of invasive disease by heterologous Nme strains containing isotype II TbpBs after immunization with LCL. Graphs showing i) mouse survival, ii) total clinical score, and iii) bacteremia after intraperitoneal injection of the indicated *N. meningitidis* strain into LCL-immunized (red) or control (grey) C57BL/6 male mice. N = 5 mice per group with each mouse depicted by a different shape. Thick solid line connects median clinical score at each time point. Dotted line at 10 shows the clinical score cutoff at which animals were humanely euthanized (panel ii) or limit of detection of bacteremia (panel iii). Two-way ANOVA with Bonferroni’s multiple comparison test comparing vaccinated versus control groups at each time point was performed on clinical scores using GraphPad Prism 10.2.0. Only p-values <0.05 indicated. *, p < 0.05; **, p < 0.01.

LCL-immunized C57BL/6 mice were intraperitoneally infected with 10^6^-10^7^ colony forming unit (CFU) of the selected Nme strains and then monitored for the progression of clinical symptoms (Fig 8), with mice reaching a clinical score of 10 being considered at clinical endpoint and humanely euthanized. Consistent with antibody coverage, clinical scores in mice immunized with LCL and challenged with the three serogroup B Isotype II Nme strains (NG144/82 (Nme 1), H44/76 (Nme 3), NGPB24 (Nme 4), having TbpBs sharing 82%, 68%, and 69% sequence identity with full-length M982 TbpB, respectively) were lower than the clinical scores for mice that received only the adjuvant control. Serum from mice that had bactericidal activity against Nme M982 shown in Fig 5E were pooled and assessed for bactericidal activity against these heterologous strains of *N. meningitidis.* While 50% killing was not detected at any dilution of serum tested, low levels of complement-mediated killing were observed by both pooled intact TbpB serum and LCL serum (S8 Fig). There was no difference in clinical outcome when mice were challenged with the Isotype I Nme strain (serogroup B strain B16B6, 37% sequence identity with M982 TbpB), consistent with the lack of antibody cross-reactivity seen.

### LCL protects against nasal colonization by a heterologous Isotype II meningococcal strain

**Fig 9:**
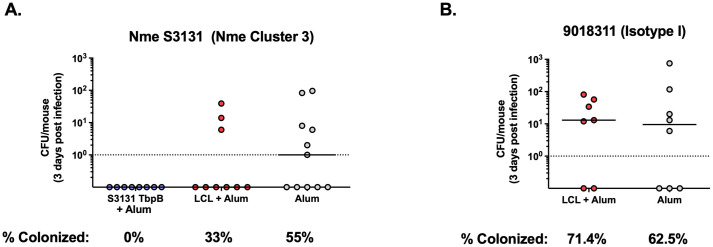
Reduction in colonization rates by a heterologous Nme strain containing an Isotype II TbpB after immunization with LCL. Meningococcal burden in the nasopharynx three days post nasal administration of A: Nme 3 Isotype II strain S3131 and B: Isotype I strain 90/18311. The percentage of culture positive animals is indicated below. N = 7-11 hCEACAM1 + /- (mCEACAM1 + /+) FvB mice in each group. Each circle represents CFU from one animal, line at median. One-way ANOVA with Dunnett’s multiple comparison of the bacterial burden in each vaccinated group to the control (A) and non-parametric Mann-Whitney test (B) performed using GraphPad Prism 10.2.0 did not yield significant p-values.

Finally, we assessed LCL-mediated cross-protection against mucosal colonization. Vaccinated hCEACAM1(+/-) mice were intranasally challenged with a serogroup B Isotype II Nme strain S3131 (Fig 9A) that expresses a heterologous TbpB (Nme 3, 69% amino acid sequence identity to M982 TbpB). Bacterial burden was quantified in nasopharyngeal tissues three days post infection, revealing the proportion of culture positive animals as the primary readout. Compared to adjuvant alone (55% or 6/11 colonized), the homologous S3131 TbpB antigen (included as a positive control) was sterilizing (0% or 0/8) against this strain. LCL immunization reduced the colonization rate compared to adjuvant alone (33% or 3/9), demonstrating its ability to provide partial mucosal cross-protection.

Since mechanisms underlying mucosal protection are not yet well-defined, despite the absence of LCL mediated antibody cross-reactivity to gonococcal TbpBs and meningococcal Isotype I TbpBs, we proceeded to evaluate nasopharyngeal protection by a TbpB Isotype I Nme strain (serogroup C strain 90/18311; 37% sequence identity) in hCEACAM1(+/-) mice and lower genital tract protection against an Ngo 1 (FA1090; ~ 60% sequence identity) and an Ngo 2 strain (FA19; ~ 68% sequence identity) in wild type female C57BL/6 mice. We found no difference in mice immunized with LCL compared to those that received the adjuvant only control (Figs 9B, S9).

Together, our cross-protection studies indicate that in addition to protecting against invasive infection, LCL may reduce colonization rates by Isotype II Nme strains.

## Discussion

The engineered LCL presented here is comprised of a minimized structure with the conserved regions of the candidate vaccine antigen, TbpB, towards the development of a broadly protective vaccine against the pathogenic Neisseria species. While originally developed as a soluble scaffold to display loops from integral membrane proteins that are otherwise challenging to target through vaccination, in this study we show that LCL alone exhibits remarkable stability and functionality as an independent immunogen, suggesting its potential as a versatile tool for vaccine applications.

That LCL was able to protect against homologous challenge to a similar extent as full-length TbpB was unexpected. Our earlier studies comparing full-length TbpB to individual lobes (Fig 3D) suggested that conformational antibodies near the junction of the two lobes may be critical since protection was lost even when individually produced lobes were combined. Furthermore, studies in pigs using engineered full-length TbpB antigens indicated that N-lobe specific antibodies that block host transferrin from binding TbpB on the surface of *G. parasuis* during infection are critical for protection. This is likely due to iron starvation caused by antibodies that target the transferrin binding site providing an added protective mechanism [34]. Therefore, the ability of LCL to retain protective function despite its significant truncation and inability to elicit antibodies that block transferrin mediated iron uptake provides new insights into protection determinants.

Protein ELISAs clearly demonstrated that anti-LCL antibodies bind the C-lobe specifically, and do not cross-react with the N-lobe, implying that effective targeting of the C-lobe alone can be sufficient to elicit protection. This raises further questions as to why immunization with LCL, but not C-lobe, was effective. One explanation could be that the increased fold stability and proteolytic resistance of LCL allows this antigen to retain its tertiary structure after immunization, eliciting robust conformational antibodies, as reported in cases of other stabilized immunogens [36,37]. Differences in performance may also lie in differences in what antibody repertoire is elicited between C-lobe and LCL. The sequence variability of the C-lobe loop regions suggest that the loops are immunogenic and have been subjected to selective pressures over time. Thus, it is reasonable to conclude that anti-C-lobe antibodies may largely be against the loop regions and thus distinct from the LCL repertoire. While beyond the scope of this study, comparison of T cell responses would also be required for fully appreciating the immunological differences.

The correlation between bactericidal activity of serum and protection from invasive disease has been clearly demonstrated in various vaccine trials, particularly for conjugate capsular serogroup C vaccines [38]. Additionally, the increased incidence of meningococcal disease in individuals with terminal complement pathway defects similarly implicates bactericidal activity as being vital for protection from these infections [39]. Despite this, in studies presented here, both full-length TbpB and LCL elicited robust protection against invasive challenge by the homologous strain, yet only some TbpB or LCL immunized mice had serum with bactericidal activity. Similarly, TbpA has been previously shown to be fully protective in mouse sepsis studies, yet lacked robust bactericidal activity [40]. Together, these data indicate that bactericidal titres may not always be predictive of protection from invasive challenge of *N. meningitidis* by protein-based vaccines and additional immunological pathways may be implicated.

Our initial hypothesis that immunization with LCL could result in broader TbpB coverage compared to the parental full-length M982 TbpB was also confirmed in this study. Anti-LCL antibody specificity for diverse Nme Isotype II TbpBs, but not Ngo TbpBs that cluster within the overall Isotype II diversity, was somewhat unexpected considering that the Ngo 2 sub-cluster branches off the larger Nme 2 sub-cluster, where M982 TbpB belongs. However, this specificity provides an opportunity for future work to identify residues that align within LCL that are unique to Nme Isotype II TbpBs and map potential cross-reactive and cross-protective B cell epitopes. This would be crucial in guiding the future design of LCL-based hybrid antigens to avoid disrupting its inherent protective capabilities.

A distinguishing feature of full-length TbpB and the LCL is the ability to protect against mucosal colonization [22,41]. Interestingly, this is not a common feature of all SLPs since immunization and challenge with homologous fHbp in this study or with 4CMenB vaccine against the fHbp-variant matched strain in a previous study [20] did not reduce meningococcal colonization rates in our hands in transgenic hCEACAM1 mice, and the 4CMenB vaccine does not protect against Nme colonization in humans [42]. This discrepancy is likely due to an interplay of both host responses to the different immunogens and bacterial responses to different environments regulating expression of these targets. The mucosal protection conferred by TbpB-specific antibodies may, therefore, result from the combination of their ability to starve the bacteria of iron and clear the bacteria by opsonization-dependent processes. As the work presented here was performed in mice lacking hTf, the contribution of iron restriction on meningococcal clearance from mucosal surfaces remains to be elucidated.

Overall, SLPs have been recognized as enticing candidate vaccine antigens for a wide variety of gram-negative pathogens. SLPs are consistently comprised of a β-barrel and a handle domain, a pattern duplicated in each lobe of TbpB. The protection shown here of a minimized version of a single lobe of TbpB offers a framework for engineering other SLPs as more broadly cross-protective antigens by removing hypervariable loops while maintaining antigen stability. As more SLPs are identified in pathogens of interest, this strategy of antigen engineering may allow for accelerated vaccine development going forward.

Together, we have demonstrated that engineering a meningococcal TbpB to a minimized, stable LCL provides a superior immunogen that elicits a broadly cross-protective immune response against Isotype II meningococcal TbpBs and presents a novel strategy towards developing broad-spectrum protein-based vaccines.

## Materials and methods

### Ethics statement

Meningococcal infection studies were performed under the animal use protocol 20011319, approved by the Animal Care Committee at the University of Toronto. Gonococcal mouse infection studies were performed under the animal use protocol 20011775, approved by the Animal Care Committee at the University of Toronto. Immune rabbit serum was collected in rabbit studies performed under protocol AC11–0033, approved by the University of Calgary Animal Care Committee.

### Protein production

For detailed expression and purification methods, see the S1 Methods. Briefly, full-length meningococcal M982 TbpB, C-lobe, and LCL were cloned into a custom T7 protein expression vector as His-tagged maltose binding protein (MBP) fusions as previously described [17]. Vectors were transformed into *E.coli* T7 express cells (New England Biolabs) and protein expression was carried out overnight at 20^o^C in autoinduction ZY media [43]. Cells were harvested by centrifugation, resuspended in lysis buffer and lysed by homogenization (EmulsiFlex-C3, Avestin). Lysate was clarified by centrifugation, followed by syringe filtration (0.22 µm), before application onto HisTrap EXCEL columns (Cytiva). Protein was eluted using imidazole and pooled fractions were dialyzed and TEV cleaved overnight. Cleaved proteins were separated by anion exchange (HiTrapQ column, Cytiva), followed by size exclusion chromatography (HiPrep 26/60 Sephracryl S-200 HR, Cytiva). A final polishing step with a strong anion exchange MonoQ 5/50 GL column (Cytiva) was used to remove lipopolysaccharides, and sample purity was assessed by 12% SDS-PAGE. Samples were concentrated and flash frozen until used.

### Crystallization of LCL

Purified LCL was initially screened with an automated Gryphon robot (Art Robbins) against Hampton and JCSG+ (Qiagen) commercial screening suites using sitting drop vapour diffusion. Initial hits were optimized with a 1:1 (protein: precipitant) ratio in a precipitant solution composed of 0.1 M Bicine pH 9.0, 20% PEG 6000 that yielded crystals that diffracted to 1.95 Å in space group P 4 21 2.

### Data collection and structure determination

Crystallographic data was collected on crystals at 80 K at the Advanced Photon Source (NECAT-24-ID-E beamline). The diffraction dataset was collected at a wavelength of 0.9795 Å and processed using DENZO and SCALEPACK from the HKL-2000 suite [44]. The first structural model for the LCL was obtained by molecular replacement using Phenix PHASER [45] with a starting model based on a C-lobe truncation from the full-length M982 TbpB crystal structure (PDB 3VE2). The final model was generated following several rounds of model building and refinement using Coot [46] and Phenix REFINE [47]. Data refinement and statistics are summarized in S1 Table.

### Accession codes

Structure factors and atomic coordinates for M982 TbpB LCL have been deposited to the PDB under accession code 5KKX.

### Nano differential scanning fluorimetry

Measurements were carried out on a Tycho NT.6 (NanoTemper). Purified protein (final concentration of 0.2 mg/mL) in either high pH (50 mM sodium phosphate, 150 mM NaCl, pH 7.5) or low pH (50 mM sodium citrate, 150 mM NaCl, pH 4.0) buffers were loaded into a glass capillary through capillary action and placed over the instrument mirror. Measurements were conducted between 35^o^C and 95^o^C at a rate of 30^o^C/min.

### Protease stability assay

Antigen susceptibility to proteases was assessed using trypsin (Promega) and chymotrypsin (Bioshop) at protease:antigen ratios of 1:1,000 and 1:100 respectively. A 60 µL solution of 0.2 mg/mL purified protein was co-incubated with either protease at 37^o^C for up to 3.5 h, taking samples at indicated time points. For each time point, 10 µL of reaction was quenched by the addition of 5 mM PMSF, mixed with 10 µL of SDS loading buffer, and boiled for 5 min. Samples were run on a 15% SDS-PAGE gel and underwent densitometry analysis using ImageJ [48].

### Mouse studies

For all mouse studies, mice were kept under specific pathogen free conditions and given access to food and water *ad libitum.* Required group sizes were estimated using a Type I error rate of 0.05 and 80% power, with expected protection of 70% of the group, leading to initial group sizes of 5 mice/group. Group sizes were reduced in some sepsis studies, as consistent data was seen for naïve or adjuvant-only groups and full protection was observed with protective antigens. This reduction was in line with our institutional ‘Reduction Goals in Animal Studies’. Group sizes for meningococcal colonization studies vary slightly due to number and genotype of transgenic human CEACAM1 mice born, with replicate studies performed to confirm reproducibility and increase the confidence of the data.

### Mouse immunizations

Purified protein was first diluted in phosphate buffered saline (PBS) and then mixed with 2% Alhydrogel to a final concentration of 25 μg protein and 100 μg Alum per 100 μL dose and allowed to adsorb for at least 20 mins with gentle agitation. For some studies, Emulsigen-D (MVP adjuvants) ready-to-use emulsion was added to diluted protein to a final concentration of 20% (v/v). Vaccines were administered on days 0, 21, and 42 via intraperitoneal injection, unless otherwise specified in the figure legend. For passive immunizations, terminal serum from a New Zealand White rabbit immunized 3 times subcutaneously with 50 μg of LCL with 20% (v/v) Emulsigen-D was obtained from the Schryvers lab was heat inactivated at 56^o^C for 30 minutes and 250 µL was injected intraperitoneally 6 hours prior to infection.

### Invasive meningococcal mouse infections

Invasive sepsis challenge experiments were performed as previously described [17,49]. Clinical symptoms were scored at regular intervals, with assessments including dehydration, movement changes, mouse grimace score, occurrence of diarrhea, posture, and changes in respiration. A cumulative score of 10 or a weight loss of 20% was used as the clinical cutoff for humane euthanasia by cervical dislocation. For detailed infection methodology, see S2 Methods.

### Meningococcal nasopharyngeal colonization

Nasopharyngeal colonization experiments were performed as previously described [32,41]. For detailed infection methodology, see S3 Methods.

### Gonococcal lower genital tract colonization

Lower genital tract colonization experiments were performed as previously described [17,41]. For detailed infection methodology, see S4 Methods.

### ELISAs

ELISAs were performed as previously described [17,41]. For detailed immunogenicity methods, see S5 Methods.

### Serum bactericidal assays

Serum bactericidal assays were performed as previously described [41]. Briefly, Nme M982 was grown overnight on GC-IsoVitaleX plates at 37°C with 5% CO_2_. The next day, bacteria was restreaked onto fresh GC-IsoVitaleX plates containing 100 mol/L deferoxamine mesylate salt (Sigma, cat. D9533) for 4 h. Bacteria were collected off the plates using a Dacron swab, resuspended in PBS++, and OD measured. Assay was set up in 40 μL total volume using ~500 bacteria suspended in PBS++, 10% baby rabbit complement (Cedarlane, CL3441-S100) and 2-fold serial dilutions of heat inactivated terminal mouse serum. The dilution at which 50% killing was observed relative to no antibody control (rabbit complement only) in 60 minutes was reported as the SBA titre. To reflect the potential over estimate of bactericidal activity when using rabbit complement, we have considered 50% killing at a 1:8 serum dilution as the cut off for considering a serum sample bactericidal, as previously described [33].

### hTf growth inhibition assay

Nme M982 was streaked onto GC-Kellogg’s plates and grown overnight at 37^o^C with 5% CO_2_. Bacteria were collected with a Dracon swab and resuspended into RPMI 1640 (Sigma, cat. R8758) to an OD_600_ of 0.075 and grown at 37^o^C with shaking at 160 rpm for 4 hours. Serum from mice immunized with their respective antigen was pooled and heat-inactivated at 56^o^C for 40 minutes and filtered (0.22 µm filter, Corning Costar, cat. CLS8161) to remove any aggregates and contaminants. In a 96-well round bottom plate, sera were diluted 1:20 in RPMI 1640 supplemented with a final concentration of 0.14 μM (10 μg/mL) of holo hTf (Sigma, cat no. T4132) to approximately an OD_600_ of 0.2. Bacterial growth was measured through OD_600_ using a Cytation 5 cell imaging multimode reader (BioTek) every 4 minutes for 10 hours at 37^o^C with shaking every 20 seconds.

### Western blots

Bacterial strains were grown overnight on GC-IsoVitaleX plates at 37°C with 5% CO_2_, followed by an additional subculture on GC-IsoVitaleX containing 100 µM deferoxamine mesylate salt for 4 hours to induce TbpB expression. Whole cell lysates were prepared as described previously [22]. Samples were resolved on 10% SDS–PAGE and transferred to PVDF membranes. Membranes were blocked in 5% skim milk in Tris-buffered saline with 0.05% Tween (TBST) for 1 h, followed by overnight incubation at 4°C with gentle agitation in 1:5,000 dilution of either pooled mouse or rabbit serum in 5% skim milk. Secondary antibody (either goat-anti-mouse IgG or goat anti-rabbit IgG, Jackson Immuno Research, cat. 115-035-003 or 111-035-144) was used at 1:15,000 dilution in 5% skim milk for 2 h at room temperature, after which Novex ECL Chemiluminescent Substrate Reagent Kit (Invitrogen) was added for band detection.

## Supporting information

S1 Fig Protease stability experiments. Representative gels for protease stability experiments are shown for A: full-length TbpB, B: C-lobe, and C: LCL. Samples of 0.2 mg/mL purified protein were co-incubated with trypsin or chymotrypsin (at ratios of 1:1,000 and 1:100 protein:enzyme, respectively) and allowed to react at 37^o^C for up to 3.5 h, taking samples at indicated time points. Densitometry was performed (n = 3) using ImageJ.
(TIFF)

S2 Fig Comparison of TbpB versus individual N- and C-lobes or fHbp as vaccine antigens. A: Graph depicting meningococcal burden in the nasopharynx of immunized hCEACAM1 + /- (mCEACAM1-/-) C57BL/6 mice three days post nasal administration of the homologous M982 Nme strain, with the percentage of culture positive animals indicated below. B: Serum bactericidal titre against iron-starved Nme M982 using terminal serum from mice from panel B. N = 12 for adjuvant control group, N = 11 for others. A: Dotted lines represent lower limit of detection. Each symbol represents one animal, line at median. One-way ANOVA with Tukey’s post-hoc test comparing each group to every other group performed, p < 0.05 indicated. For B, log2 transformed data was used for statistical analysis. *, p < 0.05; **, p < 0.01.
(TIFF)

S3 Fig Comparison of anti-TbpB IgG subclasses, IgM, and IgA elicited by TbpB, C-Lobe, and LCL antigens. Protein ELISA measuring reactivity of TbpB, C-lobe, and LCL-immune sera against full-length TbpB, evaluating A: total IgG, B: IgG1, C: IgG2a, D: IgG2b, E: IgG3, F: IgM, and G: IgA. Serum from terminal samples of hCEACAM1 FvB mice immunized three times with the indicated antigen via intra-peritoneal injection, followed by nasal infection of *N. meningitidis* strain M982, with samples collected three days post infection. Data represents samples from four different infection studies, with total number of samples of N = 25 for intact TbpB, N = 19 for C-lobe, N = 23 for LCL, and N = 17 for alum, with each sample tested in duplicate and the average interpolated value (panels A, F, G) or OD450 value (panels B-E) shown. H: Protein ELISA measuring reactivity of nasal lavage samples from TbpB, C-lobe, and LCL-immunized mice against full-length TbpB, evaluating i) IgG, ii) IgM, and iii) IgA. Samples from one of the four nasal infection studies from panels A-G, with data representing interpolated values from undiluted nasal lavage samples tested in duplicate for N = 5–8 mice per group. Error bars represent mean + /- standard deviation. One-way ANOVA with Tukey’s post-hoc test comparing each group to every other group performed using GraphPad Prism 10.2.0. ns, not significant; *, p < 0.05; **, p < 0.01; ***, p < 0.001; ****, p < 0.0001.
(TIFF)

S4 Fig Sequence alignment of TbpB C-lobes. Protein sequence alignment of the TbpB C-lobes for the 17 strains tested in Fig 6. The bar graph below the alignment displays the Quality for each alignment column, which is a measure of sequence conservation. Figure generated using Jalview v2.11.5.1 [50].
(PDF)

S5 Fig Immunoblots depicting TbpB specificity. Anti-TbpB and anti-LCL mouse serum (pooled from 8 animals per group) and anti-LCL rabbit serum (from a single animal) were used to probe whole-cell lysates prepared from iron-starved meningococcal and gonococcal strains indicated above.
(PNG)

S6 Fig Whole cell ELISA confirmation of TbpB expression under iron restriction. Serum reactivity of pooled serum from intact TbpB, C-lobe, LCL, or adjuvant only immunized mice (N = 4 mice per group pooled) tested against a subset of *N. meningitidis* strains of interest at 1:4,000 dilution or HRP-labelled human transferrin (hTf-HRP, 1:500 dilution), tested in technical quadruplicate. *N. meningitidis* strains were grown on rich media (Chocolate agar) or under iron starvation (two overnight growths on GC supplemented with IsoVitaleX and 10 μM deferoxamine mesylate salt) to confirm iron restriction leads to TbpB expression, heat killed, and then dried on ELISA plates prior to evaluation. Error bars represent mean + /- standard deviation. Two-way ANOVA with Sidak’s multiple comparisons test comparing the two growth conditions performed using GraphPad Prism 10.2.0. ns, not significant; ****, p < 0.0001.
(TIFF)

S7 Fig Whole cell ELISA immunogenicity of intact, C-lobe, and LCL serum. Serum reactivity of pooled serum from intact TbpB, C-lobe, LCL, or adjuvant only immunized mice (N = 4 mice per group pooled) tested against a subset of *N. meningitidis* strains of interest at dilutions from 1:500–1:8,000, tested in technical quadruplicate. Error bars represent mean + /- standard deviation. *N. meningitidis* strains were grown under iron restriction, heat killed, and then dried on ELISA plates prior to evaluation.
(TIFF)

S8 Fig Serum bactericidal killing of heterologous meningococcal strains. Serum from mice that showed bactericidal activity against the homologous strain M982 were pooled and tested for bactericidal killing against three different meningococcal strains. A: Pooled serum was re-tested against M982 to ensure bactericidal killing was seen, and then tested against B: NG144/82, C: H44/76, and D: NGPB24. Each circle represents one biological replicate. Percent killing is plotted relative to time zero. Line depicts mean.
(TIFF)

S9 Fig Lower genital tract colonization of female mice by representative heterologous Ngo strains. Ai, Bi: Graphs depicting % of animals that remain colonized over a three week period after vaginal inoculation with FA1090 and FA19, respectively. Groups sizes and median colonization duration indicated below. Log-rank (Mantel-Cox test) comparing Alum versus LCL curves did not yield significant p-values. Bi, Bii: Total bacterial burden of FA1090 and FA19 infected animals respectively, calculated using area under the curve (AUC) by first plotting daily CFU recovered from vaginal lavages. N = 14–17 per group. Line at median. Non-parametric Mann-Whitney test did not yield significant p-values. Statistical analysis performed using GraphPad Prism 10.2.0.
(TIFF)

S1 Table Refinement statistics for the LCL crystal structure. (PDF)

S1 Methods Protein Production. (DOCX)

S2 Methods Invasive Meningococcal Mouse Infections. (DOCX)

S3 Methods Meningococcal Nasopharyngeal Colonization. (DOCX)

S4 Methods Gonococcal Lower Genital Tract Colonization. (DOCX)

S5 Methods ELISAs. (DOCX)

S1 Data Additional data for data availability available in S1_Data.xlsx for the following experiments: Recovered CFUs from individual mouse from challenge studies. Individual mouse clinical scores from invasive challenge studies. Serum bactericidal titres for mice for homologous SBA studies
(XLSX)

S2 Data File ‘S2_Data.txt’ includes protein sequences for the C-lobes of interest in TbpBs studied here. (TXT)

## References

[1] Voß F, Kohler TP, Meyer T, Abdullah MR, van Opzeeland FJ, Saleh M, et al. Intranasal Vaccination With Lipoproteins Confers Protection Against Pneumococcal Colonisation. Front Immunol. 2018;9:2405. doi: 10.3389/fimmu.2018.02405 30405609 PMC6202950

[2] Steere AC, Sikand VK, Meurice F, Parenti DL, Fikrig E, Schoen RT, et al. Vaccination against Lyme disease with recombinant Borrelia burgdorferi outer-surface lipoprotein A with adjuvant. Lyme Disease Vaccine Study Group. N Engl J Med. 1998;339(4):209–15. doi: 10.1056/NEJM199807233390401 9673298

[3] Seib KL, Scarselli M, Comanducci M, Toneatto D, Masignani V. Neisseria meningitidis factor H-binding protein fHbp: a key virulence factor and vaccine antigen. Expert Rev Vaccines. 2015;14(6):841–59. doi: 10.1586/14760584.2015.1016915 25704037

[4] Rokbi B, Mignon M, Maitre-Wilmotte G, Lissolo L, Danve B, Caugant DA, et al. Evaluation of recombinant transferrin-binding protein B variants from Neisseria meningitidis for their ability to induce cross-reactive and bactericidal antibodies against a genetically diverse collection of serogroup B strains. Infect Immun. 1997;65(1):55–63. doi: 10.1128/iai.65.1.55-63.1997 8975892 PMC174556

[5] Lissolo L, Maitre-Wilmotte G, Dumas P, Mignon M, Danve B, Quentin-Millet MJ. Evaluation of transferrin-binding protein 2 within the transferrin-binding protein complex as a potential antigen for future meningococcal vaccines. Infect Immun. 1995;63(3):884–90. doi: 10.1128/iai.63.3.884-890.1995 7868259 PMC173085

[6] Price GA, Russell MW, Cornelissen CN. Intranasal administration of recombinant Neisseria gonorrhoeae transferrin binding proteins A and B conjugated to the cholera toxin B subunit induces systemic and vaginal antibodies in mice. Infect Immun. 2005;73(7):3945–53. doi: 10.1128/IAI.73.7.3945-3953.2005 15972481 PMC1168620

[7] Hooda Y, Shin HE, Bateman TJ, Moraes TF. Neisserial surface lipoproteins: structure, function and biogenesis. Pathog Dis. 2017;75(2):10.1093/femspd/ftx010. doi: 10.1093/femspd/ftx010 28158534

[8] Adamiak P, Calmettes C, Moraes TF, Schryvers AB. Patterns of structural and sequence variation within isotype lineages of the Neisseria meningitidis transferrin receptor system. Microbiologyopen. 2015;4(3):491–504. doi: 10.1002/mbo3.254 25800619 PMC4475390

[9] Gandhi A, Balmer P, York LJ. Characteristics of a new meningococcal serogroup B vaccine, bivalent rLP2086 (MenB-FHbp; Trumenba®). Postgrad Med. 2016;128(6):548–56. doi: 10.1080/00325481.2016.1203238 27467048

[10] Serruto D, Bottomley MJ, Ram S, Giuliani MM, Rappuoli R. The new multicomponent vaccine against meningococcal serogroup B, 4CMenB: immunological, functional and structural characterization of the antigens. Vaccine. 2012;30 Suppl 2(0 2):B87-97. doi: 10.1016/j.vaccine.2012.01.033 22607904 PMC3360877

[11] Pogoutse AK, Moraes TF. Iron acquisition through the bacterial transferrin receptor. Crit Rev Biochem Mol Biol. 2017;52(3):314–26. doi: 10.1080/10409238.2017.1293606 28276700

[12] Echenique-Rivera H, Muzzi A, Del Tordello E, Seib KL, Francois P, Rappuoli R, et al. Transcriptome analysis of Neisseria meningitidis in human whole blood and mutagenesis studies identify virulence factors involved in blood survival. PLoS Pathog. 2011;7(5):e1002027. doi: 10.1371/journal.ppat.1002027 21589640 PMC3088726

[13] Cornelissen CN, Kelley M, Hobbs MM, Anderson JE, Cannon JG, Cohen MS, et al. The transferrin receptor expressed by gonococcal strain FA1090 is required for the experimental infection of human male volunteers. Mol Microbiol. 1998;27(3):611–6. doi: 10.1046/j.1365-2958.1998.00710.x 9489672

[14] Agarwal S, King CA, Klein EK, Soper DE, Rice PA, Wetzler LM, et al. The gonococcal Fur-regulated tbpA and tbpB genes are expressed during natural mucosal gonococcal infection. Infect Immun. 2005;73(7):4281–7. doi: 10.1128/IAI.73.7.4281-4287.2005 15972520 PMC1168583

[15] Retzer MD, Yu R h, Zhang Y, Gonzalez GC, Schryvers AB. Discrimination between apo and iron-loaded forms of transferrin by transferrin binding protein B and its N-terminal subfragment. Microb Pathog. 1998;25(4):175–80. doi: 10.1006/mpat.1998.0226 9817820

[16] Noto JM, Cornelissen CN. Identification of TbpA residues required for transferrin-iron utilization by Neisseria gonorrhoeae. Infect Immun. 2008;76(5):1960–9. doi: 10.1128/IAI.00020-08 18347046 PMC2346694

[17] Fegan JE, Calmettes C, Islam EA, Ahn SK, Chaudhuri S, Yu R-H, et al. Utility of Hybrid Transferrin Binding Protein Antigens for Protection Against Pathogenic Neisseria Species. Front Immunol. 2019;10:247. doi: 10.3389/fimmu.2019.00247 30837995 PMC6389628

[18] Marshall HS, McMillan M, Koehler AP, Lawrence A, Sullivan TR, MacLennan JM, et al. Meningococcal B Vaccine and Meningococcal Carriage in Adolescents in Australia. New England Journal of Medicine. 2020;382(4):318–27. 10.1056/NEJMoa1900236 31971677

[19] McMillan M, Chandrakumar A, Wang HLR, Clarke M, Sullivan TR, Andrews RM, et al. Effectiveness of Meningococcal Vaccines at Reducing Invasive Meningococcal Disease and Pharyngeal Neisseria meningitidis Carriage: A Systematic Review and Meta-analysis. Clin Infect Dis. 2021;73(3):e609–19. doi: 10.1093/cid/ciaa1733 33212510

[20] Buckwalter CM, Currie EG, Tsang RSW, Gray-Owen SD. Discordant Effects of Licensed Meningococcal Serogroup B Vaccination on Invasive Disease and Nasal Colonization in a Humanized Mouse Model. J Infect Dis. 2017;215(10):1590–8. doi: 10.1093/infdis/jix162 28368526 PMC5461428

[21] Rokbi B, Renauld-Mongenie G, Mignon M, Danve B, Poncet D, Chabanel C, et al. Allelic diversity of the two transferrin binding protein B gene isotypes among a collection of Neisseria meningitidis strains representative of serogroup B disease: implication for the composition of a recombinant TbpB-based vaccine. Infect Immun. 2000;68(9):4938–47. doi: 10.1128/IAI.68.9.4938-4947.2000 10948108 PMC101705

[22] Fegan JE, Islam EA, Curran DM, Ng D, Au NYT, Currie EG, et al. Rational selection of TbpB variants yields a bivalent vaccine with broad coverage against Neisseria gonorrhoeae. NPJ Vaccines. 2025;10(1):10. doi: 10.1038/s41541-024-01054-0 39814726 PMC11736018

[23] Ling JML, Shima CH, Schriemer DC, Schryvers AB. Delineating the regions of human transferrin involved in interactions with transferrin binding protein B from Neisseria meningitidis. Mol Microbiol. 2010;77(5):1301–14. doi: 10.1111/j.1365-2958.2010.07289.x 20633231

[24] Calmettes C, Alcantara J, Yu R-H, Schryvers AB, Moraes TF. The structural basis of transferrin sequestration by transferrin-binding protein B. Nat Struct Mol Biol. 2012;19(3):358–60. doi: 10.1038/nsmb.2251 22343719 PMC3981719

[25] Moraes TF, Yu R, Strynadka NCJ, Schryvers AB. Insights into the bacterial transferrin receptor: the structure of transferrin-binding protein B from Actinobacillus pleuropneumoniae. Mol Cell. 2009;35(4):523–33. doi: 10.1016/j.molcel.2009.06.029 19716795

[26] Qamsari MM, Rasooli I, Chaudhuri S, Astaneh SDA, Schryvers AB. Hybrid Antigens Expressing Surface Loops of ZnuD From Acinetobacter baumannii Is Capable of Inducing Protection Against Infection. Front Immunol. 2020;11:158. doi: 10.3389/fimmu.2020.00158 32117294 PMC7025491

[27] Ferguson MR, Delgado KN, McBride S, Orbe IC, La Vake CJ, Caimano MJ, et al. Use of Epivolve phage display to generate a monoclonal antibody with opsonic activity directed against a subdominant epitope on extracellular loop 4 of Treponema pallidum BamA (TP0326). Front Immunol. 2023;14:1222267. doi: 10.3389/fimmu.2023.1222267 37675118 PMC10478084

[28] Abramson J, Adler J, Dunger J, Evans R, Green T, Pritzel A, et al. Accurate structure prediction of biomolecular interactions with AlphaFold 3. Nature. 2024;630(8016):493–500. doi: 10.1038/s41586-024-07487-w 38718835 PMC11168924

[29] Yariv B, Yariv E, Kessel A, Masrati G, Chorin AB, Martz E, et al. Using evolutionary data to make sense of macromolecules with a “face-lifted” ConSurf. Protein Sci. 2023;32(3):e4582. doi: 10.1002/pro.4582 36718848 PMC9942591

[30] Magnusson AO, Szekrenyi A, Joosten H-J, Finnigan J, Charnock S, Fessner W-D. nanoDSF as screening tool for enzyme libraries and biotechnology development. FEBS J. 2019;286(1):184–204. doi: 10.1111/febs.14696 30414312 PMC7379660

[31] Barasuol BM, Guizzo JA, Fegan JE, Martínez-Martínez S, Rodríguez-Ferri EF, Gutiérrez-Martín CB, et al. New insights about functional and cross-reactive properties of antibodies generated against recombinant TbpBs of Haemophilus parasuis. Sci Rep. 2017;7(1):10377. doi: 10.1038/s41598-017-10627-0 28871190 PMC5583350

[32] Johswich KO, McCaw SE, Islam E, Sintsova A, Gu A, Shively JE, et al. In vivo adaptation and persistence of Neisseria meningitidis within the nasopharyngeal mucosa. PLoS Pathog. 2013;9(7):e1003509. doi: 10.1371/journal.ppat.1003509 23935487 PMC3723569

[33] Borrow R, Balmer P, Miller E. Meningococcal surrogates of protection--serum bactericidal antibody activity. Vaccine. 2005;23(17–18):2222–7. doi: 10.1016/j.vaccine.2005.01.051 15755600

[34] Frandoloso R, Martínez-Martínez S, Calmettes C, Fegan J, Costa E, Curran D, et al. Nonbinding site-directed mutants of transferrin binding protein B exhibit enhanced immunogenicity and protective capabilities. Infect Immun. 2015;83(3):1030–8. doi: 10.1128/IAI.02572-14 25547790 PMC4333475

[35] Fegan JE, Yu R-H, Islam EA, Schryvers AB. Development of a non-biased, high-throughput ELISA for the rapid evaluation of immunogenicity and cross-reactivity. J Immunol Methods. 2021;493:113037. doi: 10.1016/j.jim.2021.113037 33722512 PMC8107143

[36] Joyce MG, Zhang B, Ou L, Chen M, Chuang G-Y, Druz A, et al. Iterative structure-based improvement of a fusion-glycoprotein vaccine against RSV. Nat Struct Mol Biol. 2016;23(9):811–20. doi: 10.1038/nsmb.3267 27478931 PMC5016229

[37] Delamarre L, Couture R, Mellman I, Trombetta ES. Enhancing immunogenicity by limiting susceptibility to lysosomal proteolysis. J Exp Med. 2006;203(9):2049–55. doi: 10.1084/jem.20052442 16908625 PMC2118388

[38] Goldschneider I, Gotschlich EC, Artenstein MS. Human immunity to the meningococcus. I. The role of humoral antibodies. J Exp Med. 1969;129(6):1307–26. doi: 10.1084/jem.129.6.1307 4977280 PMC2138650

[39] Figueroa J, Andreoni J, Densen P. Complement deficiency states and meningococcal disease. Immunol Res. 1993;12(3):295–311. doi: 10.1007/BF02918259 8288947

[40] West D, Reddin K, Matheson M, Heath R, Funnell S, Hudson M, et al. Recombinant Neisseria meningitidis transferrin binding protein A protects against experimental meningococcal infection. Infect Immun. 2001;69(3):1561–7. doi: 10.1128/IAI.69.3.1561-1567.2001 11179327 PMC98056

[41] Islam EA, Fegan JE, Zeppa JJ, Ahn SK, Ng D, Currie EG, et al. Adjuvant-dependent impacts on vaccine-induced humoral responses and protection in preclinical models of nasal and genital colonization by pathogenic Neisseria. Vaccine. 2025;48:126709. doi: 10.1016/j.vaccine.2025.126709 39817984 PMC13200691

[42] McMillan M, Marshall HS, Richmond P. 4CMenB vaccine and its role in preventing transmission and inducing herd immunity. Expert Rev Vaccines. 2022;21(1):103–14. doi: 10.1080/14760584.2022.2003708 34747302

[43] Studier FW. Stable expression clones and auto-induction for protein production in E. coli. Methods Mol Biol. 2014;1091:17–32. doi: 10.1007/978-1-62703-691-7_2 24203322

[44] Otwinowski Z, Minor W. Processing of X-ray diffraction data collected in oscillation mode. Methods Enzymol. 1997;276:307–26. doi: 10.1016/S0076-6879(97)76066-X 27754618

[45] Bunkóczi G, Echols N, McCoy AJ, Oeffner RD, Adams PD, Read RJ. Phaser.MRage: automated molecular replacement. Acta Crystallogr D Biol Crystallogr. 2013;69(Pt 11):2276–86. 24189240 10.1107/S0907444913022750 PMC3817702

[46] Emsley P, Cowtan K. Coot: model-building tools for molecular graphics. Acta Crystallogr D Biol Crystallogr. 2004;60(Pt 12 Pt 1):2126–32. doi: 10.1107/S0907444904019158 15572765

[47] Adams PD, Afonine PV, Bunkóczi G, Chen VB, Davis IW, Echols N, et al. PHENIX: a comprehensive Python-based system for macromolecular structure solution. Acta Crystallogr D Biol Crystallogr. 2010;66(Pt 2):213–21. doi: 10.1107/S0907444909052925 20124702 PMC2815670

[48] Schneider CA, Rasband WS, Eliceiri KW. NIH Image to ImageJ: 25 years of image analysis. Nat Methods. 2012;9(7):671–5. doi: 10.1038/nmeth.2089 22930834 PMC5554542

[49] Johswich KO, Zhou J, Law DKS, St Michael F, McCaw SE, Jamieson FB, et al. Invasive potential of nonencapsulated disease isolates of Neisseria meningitidis. Infect Immun. 2012;80(7):2346–53. doi: 10.1128/IAI.00293-12 22508859 PMC3416473

[50] Waterhouse AM, Procter JB, Martin DM, Clamp M, Barton GJ. Jalview Version 2--a multiple sequence alignment editor and analysis workbench. Bioinformatics. 2009;25(9):1189–91. 19151095 10.1093/bioinformatics/btp033 PMC2672624

