# Supplementary material for: Rationally designed minimized TbpB confers broad protection against meningococcal infection
Source: PLoS Pathog. 2026 Aug 10;22(8):e1014493. doi: 10.1371/journal.ppat.1014493 (PMC13480600; doi:10.1371/journal.ppat.1014493)
Supplement: Supplementary file 1 [file ppat.1014493.s004.pdf]

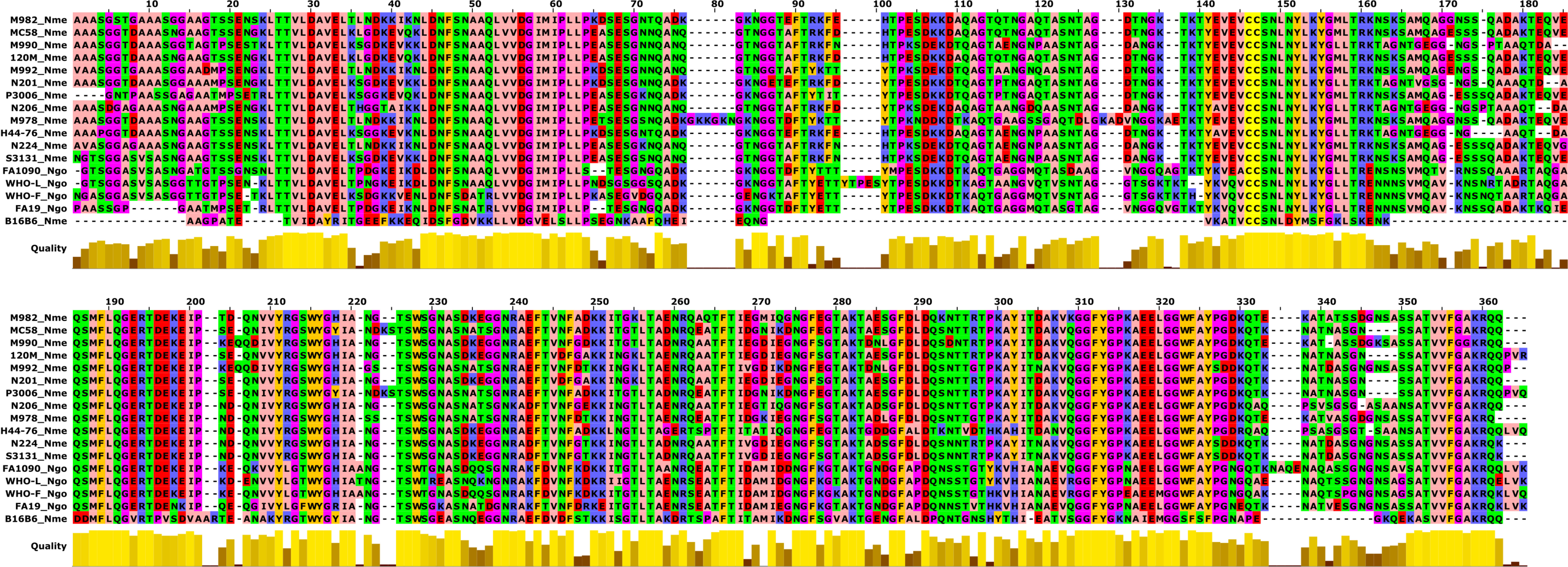
M982_Nme
MC58_Nme
M990_Nme
120M_Nme
M992_Nme
N201_Nme
P3006_Nme
N206_Nme
M978_Nme
H44-76_Nme
N224_Nme
S3131_Nme
FA1090_Ngo
WHO-L_Ngo
WHO-F_Ngo
FA19_Ngo
B16B6_Nme
Quality
