# Supplementary material for: Rationally designed minimized TbpB confers broad protection against meningococcal infection
Source: PLoS Pathog. 2026 Aug 10;22(8):e1014493. doi: 10.1371/journal.ppat.1014493 (PMC13480600; doi:10.1371/journal.ppat.1014493)
Supplement: Supplementary file 2 [file ppat.1014493.s010.pdf]

S1 Table: Refinement statistics for the LCL crystal structure.

	**LCL**
PDB Code	5kkx
Phasing Method	Molecular replacement
Data Collection	
Wavelength	0.9795
Resolution range	33.23 - 1.899 (1.95 - 1.9)
Space group	P 4 21 2
Cell Dimensions	
a, b, c (Å)	132.939 132.939 64.353
α, β, γ (°)	90 90 90
Unique reflections	45698 (3162)
Completeness (%)	99.37 (98.20)
Wilson B-factor	34.56
Refinement	
Resolution	2.1
Reflections used in refinement	45698 (3162)
Reflections used for R-free	1995 (138)
R-work	0.1825 (0.3598)
R-free	0.2166 (0.3954)
Number of non-hydrogen atoms	3842
macromolecules	3638
ligands	12
solvent	192
Protein residues	462
R.m.s. deviations	
RMS(bonds)	0.007
RMS(angles)	1.1
Ramachandran favored (%)	95.35
Ramachandran allowed (%)	4.65
Ramachandran outliers (%)	0
Rotamer outliers (%)	1.82
Clashscore	8.45
Average B-factor	52.34
macromolecules	52.36
ligands	68.4
solvent	50.94
