# Supplementary material for: Rationally designed minimized TbpB confers broad protection against meningococcal infection
Source: PLoS Pathog. 2026 Aug 10;22(8):e1014493. doi: 10.1371/journal.ppat.1014493 (PMC13480600; doi:10.1371/journal.ppat.1014493)
Supplement: Supplementary file 4 [file ppat.1014493.s019.pdf]

Response to Reviewers

Thank you for the comprehensive feedback on our manuscript and helpful suggestions on how to improve. We have performed several additional experiments to address some of the suggestions which have now been included in additional Supplementary Figures and have moved some of the original Supplemental data into the main text, as requested. Where noted, we have either reworded some of our conclusions or provided additional information or context, as appropriate. Please see below for a detailed response for how we have addressed each point, and where in the manuscript these changes can be found. All responses can be found in blue text.

Additional Editor Comments:

The Reviewers, who are experts in the field, felt that the finding that structure-informed engineered loopless C-lobe (LCL) variant of meningococcal Tbp elicits bactericidal antibodies with improved cross-protective potential against both homologous and heterologous strains of N. meningitidis is important. The Reviewers also appreciated 1) the structural characterisation of the LCL antigen, 2) the use of several mouse models for meningococcal infection, and 3) that the manuscript is well written. However, they raise a number of major issues that would need to be addressed, in some occasions by performing additional experiments. In particular, 1) considering the small number of mice/group from which conclusions are drawn and the frequent lack of statistical significance between the groups, many conclusions and titles need to be toned down or should be backed up by performing additional mouse experiments, 2) on a similar note, unlike what is stated in the text, Fig 5E shows that more than 40% of the mice immunised with C-lobe have developed bactericidal responses, hence the conclusion would be different if more mice were included in this particular group, 3) given the previous publications on the use of CL as a backbone for displaying epitopes from other antigens for immunisation, the novelty of the LCL scaffold data reported here should be better explained at the end of the Introduction, and 4) since SBA are important for evaluating any meningococcal vaccine, SBA titres should be determined against heterologous strains to strengthen the central claim that anti-LCL antibodies have wide cross-protective potential.

Journal Requirements:

1) We ask that a manuscript source file is provided at Revision. Please upload your manuscript file as a .doc, .docx, .rtf or .tex. If you are providing a .tex file, please upload it under the item type u2018LaTeX Source Fileu2019 and leave your .pdf version as the item type u2018Manuscriptu2019.

2) Please upload all main figures as separate Figure files in .tif or .eps format. For more information about how to convert and format your figure files please see our guidelines:
https://journals.plos.org/plospathogens/s/figures

3) We have noticed that you have uploaded Supporting Information files, but you have not included a list of legends. Please add a full list of legends for your Supporting Information files after the references list.

Apologies for this oversight, the legends were included in the original supplemental file. An updated supplemental legend list has now been added to the main document.

4) We are unable to open the following Supporting Information file: final_nme_ngo_tbpBs_common.faa. Please kindly revise as necessary and re-upload.

This has been changed to a .txt file that should be viewable in any text editor.

5) Please amend your detailed Financial Disclosure statement. This is published with the article. It must therefore be completed in full sentences and contain the exact wording you wish to be published.
State what role the funders took in the study. If the funders had no role in your study, please state: "The funders had no role in study design, data collection and analysis, decision to publish, or preparation of the manuscript.".
If you did not receive any funding for this study, please simply state: u201cThe authors received no specific funding for this work.u201d

Reviewers' Comments:

Reviewer's Responses to Questions

Part I - Summary
Please use this section to discuss strengths/weaknesses of study, novelty/significance, general execution and scholarship.

Reviewer #1: This is an interesting paper that advances the long history of Tbps as vaccine antigens. The authors have created a loopless C-lobe of TbpB that has improved vaccine properties to previous constructs. Tbps have application beyond N. meningitidis to other pathogens and so there will be considerable interest in this paper. The team have used appropriate methods for proof of concept experiments and there will require more exhaustive studies to confirm the vaccine potential but these are beyond the scope of this paper. The manuscript is clearly written and includes appropriate background and discussion.

Reviewer #2: The authors have designed a 'pared back' version of a Neisseria lipoprotein, TbpB, as a vaccine. The rationale, outlined by the authors in the Introduction, is to remove variable and structurally flexible regions of the molecule, to generate LCL. LCL lacks the N lobe of TbpB, as well as sections of the C lobe. They have then undertaken an extensive series of relevant experiments to evaluate the structure/stability and vaccine candidacy of LCL, compared with full length TbpB and the intact C lobe of TbpB only.

The results are interesting as LCL exhibits similar immunogenicity and protection as full length TbpB against the homologous protein or strain expressing the homologous protein, with more evidence of cross reactivity against heterologous proteins, providing support for their rationale.

The paper is well written, and the results/figures are generally laid out clearly but can be improved. There is some overreach in the conclusions they draw from their results.

Reviewer #3: The manuscript by Islam and colleagues reports on the vaccine potential of a “loopless C-lobe” TbpB protein from Neisseria meningitidis. Previously this group had engineered this protein, abbreviated LCL, as a backbone for displaying loops from other antigens for immunogenicity. Here they show the LCL itself is sufficient to elicit bactericidal antibodies against both homologous and heterologous strains of N. meningitidis, based on phylogenetic analysis of TbpB alleles. The N. meningitidis LCL does not cross-protect against genital infection with N. gonorrhoeae and does not elicit antibodies that recognize N. gonorrhoeae or confer bactericidal activity. A strength of the study is the discovery that LCL can serve as an immunogen, with promise as a vaccine antigen since it has greater stability and protease resistance in vitro than the intact C-loop or full-length TbpB. Another strength is the use of two different mouse models for infection by pathogenic Neisseria. However, this is tempered by the lack of novelty of the LCL system, the small number of mice per group from which conclusions are drawn, and the lack of investigation for how LCL elicits protective effects in both systemic and mucosal challenge.

Reviewer #4: This manuscript is of strong interest because it provides a careful and detailed characterization of the immune response induced by an engineered variant of the Tbp C-lobe lacking the most variable loops. The study shows that selective removal of immunodominant and highly variable regions can improve cross-protective potential, possibly by promoting antibody responses toward conserved epitopes. Importantly, structural analyses demonstrate that the overall fold of the C-lobe is preserved after loop deletion, supporting the feasibility of this rational antigen-design strategy.

Part II – Major Issues: Key Experiments Required for Acceptance
Please use this section to detail the key new experiments or modifications of existing experiments that should be absolutely required to validate study conclusions.

Generally, there should be no more than 3 such required experiments or major modifications for a "Major Revision"

recommendation. If more than 3 experiments are necessary to validate the study conclusions, then you are encouraged to recommend "Reject".

Reviewer #1: The manuscript makes a logical argument for the vaccine potential of the loopless C-lobe of Tbp. The authors acknowledge the limitation of the animal models and immunoassays but the data supports the claims presented.

- Thank you.

Reviewer #2: To strengthen the section on the cross-reactivity of anti-LCL antibodies (a central aspect of this work), SBA titers should be determined against heterologous strains. This would enhance the relevance of results and comments about strain coverage (325) even if no SBA titer is detected. SBA results are important for evaluating the candidacy of any meningococcal vaccine.

- Thank you for this suggestion, we agree that seeing heterologous bactericidal killing by anti-LCL sera would strengthen this work. There is insufficient serum remaining to test individual mouse samples against heterologous strains, so we have pooled samples from mice that showed bactericidal killing against the homologous strain (M982) and performed SBAs against M982 again (as a positive control), and against three different *N. meningitidis* strains of interest, NG144/82, NGPB24, and H44/76. The pooled sera did not achieve 50% killing at any of the dilutions tested, however it did display bactericidal activity. These data are now available as Supplemental Figure 8 and described in text in Lines 412-415.

Reviewer #3: 1. While the trend is promising, mouse experiments are insufficiently powered to make firm conclusions. For example, Figure 3 uses 3-4 mice per group, Figure 4 5-8 mice per group. Power calculations should be provided in Methods.

- Power calculations and group size rational has been provided in the Methods section in Lines 575-582.

2. In Figure 5, serum samples from different mouse strain backgrounds and immunization methods should not be grouped together for statistical analysis.

- Thank you for pointing this out, panels A through D have now been separated out into two plots for each ELISA, with panels 'i' showing serum from FvB mice while panels 'ii' show serum from C57Bl/6 mice and the statistical analysis has been redone for each data set.

3. Given the variability in response for individual mice, it is worth exploring the immune responses in those mice that were protected compared with those that were not. For instance, what regions of TbpB are targets of the antibodies elicited by LCL? When compared with full-length TbpB or C-lobe, this information would reveal protective antigenic regions of TbpB to refine it as a vaccine antigen. Also, are there differences in the IgG type, or in IgM or IgA response that is elicited? These analyses would greatly increase novelty of the findings.

- We agree with the reviewer that it would be very interesting in determining specific regions of LCL that lead to protection. It is clearly beyond the scope of this manuscript to do extensive epitope mapping of LCL. However, in the context of understanding the response, we performed serological analysis of serum IgG isotype, IgM, and IgA, as well as levels of anti-TbpB IgG, IgM, and IgA in nasal lavages from mice immunized with either intact TbpB, the C-lobe, or the LCL. While no substantial differences were identified in subclass or IgM/A, we did note the induction of IgG1, IgG2a, and IgG2b in serum, and IgG in mucosal lavages. Levels of IgG1, IgG2a, and IgG2b reflect overall levels of IgG, however alterations in adjuvant formulation may aid in skewing the levels of these isotypes. These new data are available as Supplemental Figure 3. These data are described in text in Lines 278-281.

4. For strains that were heterologously protected by LCL, provide a sequence alignment of their TbpB C-lobe to NmeM982, since the phylogenetic tree in Fig. 6A is for the entire TbpB protein.

- Thank you, this is an important request. The C-lobes of all TbpB sequences utilized in this study have been aligned and are available as Supplemental Figure 4.

5. Experiments examining antibody reactivity in serum of mice immunized with different TbpB-based antigens need to show that TbpB is induced in the strains tested. A M982 delta TbpB control needs to be included as well, since the parents of the two delta TbpB strains used in Fig. S2, FA19 and B1686, do not react with the immunized serum.

- Thank you for this comment. While we do not have an M982 strain lacking TbpB available for these studies, we have included a whole cell ELISA of seven strains of *N. meningitidis* grown under rich media conditions compared to under the iron starvation conditions used for these studies, showing that there is both an increase in TbpB reactivity (as seen by pooled intact TbpB serum, pooled C-lobe serum, pooled LCL serum) for all strains other than B16B6, and an increase in HRP-labelled human transferrin binding for all strains including B16B6, which confirms receptor expression. No change in reactivity was seen for pooled alum-only serum. These new data are available as Supplemental Figure 6 and described in text in Lines 358-360.

Reviewer #4: The rationale for the choice of the allele expressed and the challenge strain should be clearly stated in the first paragraphs.

- Thank you, a sentence to reflect this decision has been added in the introduction (Lines 122-124).

Figure S1A contains important information and would be more appropriately placed in the main text. In addition, the rationale for the use of different adjuvants should be clarified, including whether differences in the immune response were expected or observed. The reduced protection observed when the two lobes were used in combination also deserves further explanation.

- Thank you for this comment. The original Supplemental Figure 1A has now been added as additional panel as Figure 3D. The reduced protection observed with the two lobes mixed but not physically connected has been perplexing to us as well; however, the phenomenon is consistent to our observations with a TbpB-based porcine pathogen vaccine, where the mixture of N and C-lobes from *Glasserella parasuis* also failed to reproduce the protection provided by the full length protein. We have hypothesized that epitopes spanning the two lobes, and thus only present when the protein is in one construct, are important for full-length TbpB protection, however understanding the mechanism of this lack of protection is beyond the scope of this current manuscript. There was a sentence in the discussion raising this possibility (Lines 464-467), however we have now added an additional line has been added to the results section to further highlight this unexpected finding (Lines 199-205).

The number of mice used in the two challenge experiments (Figure 3 and S1) differs. Was variability observed in previous experiments considered when selecting only four mice for the experiment shown in Figure 3?
While the use of different mice genotypes to assess immunogenicity and protection is understandable, the rationale for selecting these specific mouse models for the different immunization routes is not explained. In particular, clarification is needed to why both hCEACAM1+/– (mCEACAM1+/+) FvB mice and hCEACAM1+/– (mCEACAM1–/–) C57BL/6 mice were included, and what specific biological question this comparison was designed to address .

- For sepsis studies with *N. meningitidis*, early studies were performed with n=5 mice per group, however as we saw consistent data for naïve or adjuvant immunized mice with M982 and full protection with “protective antigens” (full length TbpB and fHbp) in line with both literature and previous studies in our hands, we reduced the number of mice per group for subsequent studies to n=4 per group in line with our institutions’ ‘Reduction Goals in Animal Studies’. For colonization studies where mice are bred in house, but data are consistently more variable, the number of mice per group has varied based on the number and genotype of animals born. Repeat studies with comparatively low numbers of mice per group were performed confirm reproducibility and increase confidence. Similarly, repeat studies with additional routes of immunization and two transgenic lines were performed to increase confidence in the data despite the comparatively low

number of animals used in individual studies. The methods section has been updated with additional context for the group sizes used within these studies (Lines 575-582).

Line 251: The rationale for using pre-immune and post-immune sera derived from different experiments should also be clarified.

- ➢ Thank you, the use of serum from the two studies has been better contextualized, including the advantages to testing both as well as the limitations (Lines 269-273).

Line 268: The authors state that the C-lobe induces no bactericidal activity; however, some mice appear to have developed bactericidal responses. This statement should therefore be reconsidered or better contextualized.

- ➢ Thank you, this is a fair point. The language has been softened to reflect that the C lobe offered reduced, but not absent, bactericidal titres (Line 292)

The authors also mention a correlation between bactericidal activity and protection from sepsis. It would be important to know whether similar evidence was observed in the experiments presented here.
The evidence of cross-recognition is convincing and clearly presented. However, does ELISA reactivity correlate with functional cross-bactericidal activity?

- ➢ Unfortunately, there is not sufficient serum remaining from mice where we have sepsis challenge data to perform serum bactericidal assays, thus no direct evidence is available to compare bactericidal titre to protection from sepsis in the studies presented here. In this context, it is interesting to note that not all mice immunized with TbpB or the LCL elicit bactericidal titres, but all mice immunized with these antigens have survived invasive challenge, so it is likely that bactericidal titres against TbpB underestimate protection from sepsis. A paragraph has been added to the discussion regarding these findings (Lines 486-496).

The experiment shown in Figure 7 clearly indicates cross-protection following LCL immunization. It would be informative to report protection outcomes in terms of survival and bacterial clearance kinetics post-infection. In addition, the rationale for the bacterial dose selected for strains carrying different alleles should be provided. Finally, the protection data reported in Figure S3 would strengthen the manuscript if moved to the main text. The results of the challenge experiments should be presented in a consistent format to enable direct comparison across studies.

- ➢ Thank you for helpful the comments. We have expanded Figure 7 (now Figure 8) to include survival and bacteremia for each of the four sepsis challenge studies. Prior to any sepsis study with a new challenge strain, we perform a pilot dosing study to identify the dose that provides infection kinetics that allow humane monitoring of the mice (to allow them to be removed from the study if they reach clinical endpoint) while still aiming to reach lethality in naïve mice. This is not always fully reproduced, evident by the fact that strains H44/76 and NGPB24 did not achieve endpoint at the dose used, however it does allow us to account for marked virulence differences between strains. The strain-to-strain variability in virulence is well known, however the reason for the differences remains unknown. We have no evidence to suggest that these differences are due to the TbpB variant expressed by each strain.

  We have also moved the passive immunization protection data to the main text as suggested, where it is now Figure 7. To keep formatting consistent and experiments comparable, a panel depicting bacteremia after passive immunization and challenge has now been included (Figure 7Aiii).

Part III – Minor Issues: Editorial and Data Presentation Modifications
Please use this section for editorial suggestions as well as relatively minor modifications of existing data that would enhance clarity.

Reviewer #1: Line 182 - I suggest using "efficacy" to describe results of a human clinical trial. Here the authors describe protection against IP challenge in mice.

- Thanks, this has been reworded to 'protection elicited by'.

Line 189 - The loss of protection...

- Fixed, thank you.

Line 216-7 - This sentence is not clear

- This sentence has been edited for clarity.

Line 257 - "higher IgG titres in ..." Panels A and B do not show titres, rather they show OD at a fixed dilution of serum.

- Thanks for catching, this has been reworded to 'IgG reactivity at the tested serum dilution'.

Line 266 and following - "significant bactericidal activity...." The bactericidal activity is generally low and many animals do not respond with any bactericidal activity. The authors should make this clear. I would have like to have seen cross-strain SBA reported.

- Thank you, we have included the proportion of animals that did and did not elicit bactericidal activity to make this limitation clear in the figure caption for Figure 5 (Lines 315-317). Additionally, we performed heterologous SBAs using pooled serum from animals with bactericidal activity against the homologous strain; these new data are available as Supplemental Figure 8.

Line 304 - Are the protein ELISAs Fig 6C whole cell ELISAs or isolated recombinant Tbps?

- Figure 6C depicts protein ELISAs against isolated, recombinant TbpB proteins expressed in *E. coli* cytoplasm, while Figure 6D shows reactivity against whole cell bacteria (*Neisseria meningitidis* and *N. gonorrhoeae* strains) that have been iron starved and heat inactivated.

Fig. 6D - It is a pity this is a single dilution ELISA. Were doubling dilution ELISA performed. All the cross-strain ODs seem low and it's hard to tell if they are really much different to background. It is also a pity that strain M982 was not included as a "negative control"

- Unfortunately, due to the number of strains assessed and the amount of serum available from each mouse, testing doubling dilutions of sera was not feasible. However, the dilution used for testing (1:4,000) was chosen based on previous experience with serum reactivity against whole cell ELISAs using bacteria expressing the homologous TbpB of interest. A sentence to address this has been added in Lines 351-353. We did include M982 (first column on the left) to compare, as we expected this to be the strain with the highest reactivity as it expresses the homologous TbpB, which did occur here.

While insufficient serum was available to further evaluate different dilutions for individual mice, the small volume of available TbpB-reactive serum from different immunization groups was pooled and tested at different dilutions against 8 *N. meningitidis* strains of interest. Serum was tested from 1:500 to 1:8,000 in technical quadruplicate and is shown in Supplemental Figure 7. This is described in text in Lines 360-362.

Line 458 - efficacy = protection

- Fixed, thank you.

Line 543 - it would be helpful in include a brief description of the SBA method as this is important for this study.

- Thank you for this note, we agree and have thus moved the more fulsome description of our SBA methodology from the methods supplemental document back to the main text.

Reviewer #2: The authors should give the serogroup of strains when they are first mentioned in the text.

- Thank you, this has been added for M982 (line 119), 120M and N201 (line 343), NG144/82, H44/76, and NGPB24 (together, line 409), B16B6 (line 415-416), S3131 (line 432), and 90/18311 (lines 441-442).

The sequences deleted to generate LCL are not obvious from the structures shown in Figure 1; only one view of each protein is presented, and the deleted regions are mostly unstructured so difficult to see. The authors should include an alignment of the amino acid sequence of LCL with the intact C lobe (ie the sequence of proteins shown in Panels B and C). Some quantification of conserved/variable in the color code should be provided in the figure and/or text.

- An alignment of the LCL to the original C lobe has been added as an additional panel in Figure 1, and text has been added to better describe both the conservation colours and the new alignment in Lines 149-157.

Figure 2: the authors should provide the original images of proteolytic degradation in the supplementary information.

- An original image of each representative gel of the proteolytic degradation is now available as Supplemental Figure 1.

L176 delete enzyme.

- Done.

Text associated with Figures 4, 7 and 8, and conclusions in the Discussion: the authors need to be careful when describing their results given the lack of statistical significance between the groups in many instances. The titles of the associated sections and the conclusions need to be changed as often only trends towards protection (and not significant protection) were observed.

- Fair point. These titles have been reworded to better reflect the data.

Figure 4: what was the bacterial burden on other days? In previous work with this model, the group has assessed carriage on day 1 not day 3.

- Unfortunately, enumeration of bacteria after nasal infection is a terminal procedure, so it is not possible to test the bacterial burden multiple times from the same animal. For meningococcal colonization studies in vaccinated animals, our group has most frequently assessed protection at three days post infection, including in published studies from our group including Johswich *et al,* 2013; Pajon *et al*, 2015; and Buckwalter *et al*, 2017. We decided to continue tested vaccine-mediated protection at three days post challenge versus one day post challenge to better allow us to compare to these previous published studies.

251/252: the authors quote the wrong figures. Same errors in the legend of Figure 5.

- Figure quotes have been verified and updated with the additional figures from the revision stage, thank you.

Figure 5: results include data from two different mouse genotypes: these should be split into separate columns for clarity.

- Thank you for this suggestion. These data have been separated out as requested.

Figure 5E: Using terminal sera from mice that had been colonized (rather than pre-challenge) might result in boosting of anti-TbpB responses through carriage; this would not be seen in mice immunized with Alum alone. Therefore, part of the SBA results will reflect carriage as well as immunization. The authors should include this caveat in the Results/Discussion.

- Thank you for pointing this out. We have explored this previously and have not detected an increase in anti-TbpB titres between post-immunization (pre-challenge) and post-challenge (terminal) serum at the time points used for experimental endpoint. However, we agree that it is still fair to consider that the potential remains for there to have been a boosting effect. We have added a statement pointing out this limitation (lines 293-296).

SBA titers were determined with rabbit complement which can overestimate titers compared with human complement which has become the standard.

- We have added a statement noting this effect in text (Lines 623-626). To reflect the potential over estimation of bactericidal activity with rabbit complement, we have altered the cut off for considering sera bactericidal to 50% killing at a 1:8 dilution (1/log2 of higher than 2 as cutoff), as reflected in Figure 5E.

Reviewer #3: 1. Line 61: there is not sufficient analysis in this manuscript to state that the LCL "focuses the immune response". As written, the sentence on lines 58-62 implies there is cross-protection against N. gonorrhoeae, which needs to be clarified.

- The author summary has been reworded to remove the term 'focus' and to be more specific that while we tested gonococcal TbpBs, we did not see robust cross-reactivity or cross-protection to those variants.

2. Mouse clinical scoring system needs to be described in Materials and Methods.

- Thank you, this has been added in the methods section (Lines 595-599).

3. Reword subjective terms like "robust levels" (line 248) and "highly cross-reactive" (line 298).

- Subjective terms have been reworded.

4. Raw data in Figure 6B should be provided in a supplemental table.

- This has now been provided as a supplemental excel document.

5. Please explain why some experiments use alum and others Emulsigen-D as adjuvant.

- We have predominantly used alum in our vaccine formulations as it is more applicable for human vaccine projects, however some studies were performed with Emulsigen-D to explore whether emulsion-based adjuvants would recapitulate the protection that we saw with alum-based formulations. In order to fully utilize the data gained from the Emulsigen-D adjuvanted formulations, those data have been included here with the caveat that those formulations would not be directly applicable to humans.

Reviewer #4: (No Response)
