# Supplementary material for: Rationally designed minimized TbpB confers broad protection against meningococcal infection
Source: PLoS Pathog. 2026 Aug 10;22(8):e1014493. doi: 10.1371/journal.ppat.1014493 (PMC13480600; doi:10.1371/journal.ppat.1014493)
Supplement: Supplementary file 5 [file ppat.1014493.s018.pdf]

Response to Editorial Decision

Re: Manuscript PPATHOGENS-D-25-03220

This manuscript was first submitted to PLoS Pathogens on November 25th, 2025 as PPATHOGENS-D-25-03002, however was sent back to us on November 30th. In the rejection notice, there were editors comments included stating that the manuscript did not conform with PLoS Pathogens data sharing standards, and that a resubmitted version with updated data availability conforming to the guidance could be considered. As such, we have uploaded bactericidal and infection data as a supplemental file and have updated the data availability section, wherein we have specified that additional data will be uploaded to the repository 'ImmPort' ahead of the manuscript becoming publicly available.

We hope that this will fulfill the data sharing standards and thus this manuscript can be considered for publication in PLoS Pathogens.
