# Supplementary material for: Rationally designed minimized TbpB confers broad protection against meningococcal infection
Source: PLoS Pathog. 2026 Aug 10;22(8):e1014493. doi: 10.1371/journal.ppat.1014493 (PMC13480600; doi:10.1371/journal.ppat.1014493)
Supplement: Supplementary file 5 [file ppat.1014493.s011.docx]

**Protein production**

Full-length meningococcal M982 TbpB, C-lobe, and LCL were cloned into a custom T7 protein expression vector, downstream and in fusion with an N-terminal polyhistidine tagged maltose binding protein (MBP) followed by a TEV cleavage sequence as previously described [17]. Protein expression vectors were heat-shocked into chemical competent *E.coli* T7 express cells (New England Biolabs). The transformants were allowed to recover in LB broth for 1 hour, shaking at 175 RPM and 37°C, before selection by additional LB supplemented with ampicillin (100 µg/mL) and grown for a further 3 hours. The starter culture was then used to inoculate 6 L of autoinduction ZY media [43] with 50 µg/mL of ampicillin. The culture flasks were shaken overnight at 37°C and 175 RPM for 16 hours and a further 24 hours at 20°C. Cells were harvested by centrifugation at 5,000 x g for 30 minutes at 4°C. Cells were lysed by homogenization (EmulsiFlex-C3, Avestin) supplemented with protease inhibitor (c0mplete^TM^ Mini, Roche), lysozyme, and DNase I. The resulting lysate was clarified by centrifugation at 16,000 x g for 90 minutes at 4°C, followed by syringe filtration (0.22 µm). Clarified lysate was then recirculated by a peristaltic pump and affinity captured on HisTrap EXCEL columns (Cytiva) overnight, which was then mounted on an ÄKTA Purifier 100 (GE Healthcare). The column was washed and protein eluted by imidazole, and the eluted fraction was dialyzed overnight into a low imidazole buffer to facilitate cleavage by the addition of in-house purified TEV protease to separate the N-terminal MBP fusion partner. The cleaved protein was then exchanged into a low salt buffer and loaded onto an anion exchange HiTrapQ column (Cytiva), and the fractions containing TbpB, C-lobe or the LCL were concentrated and passed through a size exclusion column (HiPrep 26/60 Sephracryl S-200 HR, Cytiva) to further remove any contaminants. A final polishing step with a strong anion exchange MonoQ 5/50 GL column (Cytiva) was used to remove lipopolysacchrides, the final samples were concentrated, and aliquots were flash frozen by liquid nitrogen. Sample purity was assessed by 12% SDS-PAGE and protein concentration was determined by NanoDrop (ThermoFisher).
