# Supplementary material for: Rationally designed minimized TbpB confers broad protection against meningococcal infection
Source: PLoS Pathog. 2026 Aug 10;22(8):e1014493. doi: 10.1371/journal.ppat.1014493 (PMC13480600; doi:10.1371/journal.ppat.1014493)
Supplement: Supplementary file 6 [file ppat.1014493.s012.docx]

**Invasive meningococcal mouse infections**

4-6 week old male C57BL/6 mice were purchased from Charles River and vaccinated after a minimum of seven days of acclimatization to the facility. Infections were performed approximately 2 weeks after the final vaccine dose. Overnight lawn of the indicated Nme strain was grown on GC-Isovitalex (BD cat. 11875) or GC-Kellogg’s and sub-cultured in RPMI (Wisent, cat no. 350-000) for approximately 3-4 h at 37^o^C at 120 rpm. Bacteria were diluted in PBS containing calcium and magnesium (PBS++) and a 250 µL suspension containing approximately 10^6^-10^7^ CFU was intraperitoneally injected into isoflurane anaesthetized mice. 8 mg of holo hTf (Sigma, cat no. T4132) resuspended in 250 µL of PBS was administered at the same time as infection by a separate intraperitoneal injection for all studies, except Fig 3D where 2 mg of iron dextran in the same volume was used. Tail vein blood was sampled to monitor bacterial load in some studies and enumerated on GC Isovitalex with VCNT inhibitor (vancomycin, colistin, nystatin, and trimethoprim for selection of Neisseria species; BD BBL^TM^ VCNT Inhibitor (Thayer and Martin), Fisher Scientific, cat. [B12408](https://www.ncbi.nlm.nih.gov/nuccore/B12408)). Clinical symptoms were scored at regular intervals and a cumulative score of 10 or a weight loss of 20% was used as the clinical cutoff for humane euthanasia by cervical dislocation. Studies were performed under the animal use protocol 20011319, approved by the Animal Care Committee at the University of Toronto. For all mouse studies, mice were kept under specific pathogen free conditions and given access to food and water *ad libitum.*
