# Supplementary material for: Rationally designed minimized TbpB confers broad protection against meningococcal infection
Source: PLoS Pathog. 2026 Aug 10;22(8):e1014493. doi: 10.1371/journal.ppat.1014493 (PMC13480600; doi:10.1371/journal.ppat.1014493)
Supplement: Supplementary file 7 [file ppat.1014493.s013.docx]

**Meningococcal nasopharyngeal colonization**

Approximately 6 week old transgenic hCEACAM1+/- (mCEACAM1+/+) FvB and hCEACAM1+/- (mCEACAM1-/-) C57BL/6 mice bred in-house were immunized and then challenged intranasally approximately 2 weeks after the final dose. Briefly, overnight lawn of mouse passaged Nme grown on GC-Isovitalex (BD cat. 11875) at 37^o^C with 5% CO_2_ was suspended in PBS++ and approximately 10^7^ CFU in 10 μL was inoculated into the nostrils of isoflurane anaesthetized mice. Mice were humanely euthanized by CO_2_ overdose 3 days post infection. Terminal serum was collected by cardiac puncture and stored at -20$^{\circ}$C until analysis. Retrograde nasal lavages were collected by injecting 250 μL PBS++ through the trachea and out the nostrils and nasal turbinate was swabbed. Lavage and swab samples were grown on GC-IsoVitaleX-VCNT plates for 48 h at 37^o^C with 5% CO_2_ for selection and enumeration of *N. meningitidis.* Meningococcal colonization studies were performed under the animal use protocol 20011319, approved by the Animal Care Committee at the University of Toronto.
