# Supplementary material for: Rationally designed minimized TbpB confers broad protection against meningococcal infection
Source: PLoS Pathog. 2026 Aug 10;22(8):e1014493. doi: 10.1371/journal.ppat.1014493 (PMC13480600; doi:10.1371/journal.ppat.1014493)
Supplement: Supplementary file 8 [file ppat.1014493.s014.docx]

**Gonococcal lower genital tract colonization**

Approximately 4-6 week old female C57BL/6 mice were purchased from Charles River and vaccinated after a minimum of seven days of acclimatization to the facility. Approximately 2 weeks after the final dose, estrous cycle was monitored, hormones and antibiotics were administered and the mice were infected with Ngo as described previously [17, 41]. To prepare the inoculum, overnight lawns of Ngo FA1090 and FA19 grown on GC-Isovitalex were suspended in RPMI and ~5 μL containing approximately 10^7^ CFU was vaginally instilled. Bacteria were enumerated from vaginal lavage samples as previously described [17, 41] to determine colonization duration. Upon study completion, mice were humanely euthanized by CO_2_ overdose followed by cervical dislocation. Gonococcal studies were performed under the animal use protocol 20011775, approved by the Animal Care Committee at the University of Toronto.
