# Supplementary material for: Rationally designed minimized TbpB confers broad protection against meningococcal infection
Source: PLoS Pathog. 2026 Aug 10;22(8):e1014493. doi: 10.1371/journal.ppat.1014493 (PMC13480600; doi:10.1371/journal.ppat.1014493)
Supplement: Supplementary file 9 [file ppat.1014493.s015.docx]

**ELISAs**

For protein ELISAs, 384 well plates (VWR, cat. CA62409-064) were coated with TbpB as previously described [35]. For whole bacterial ELISAs, strains were grown overnight on GC-Isovitalex containing 10 µM deferoxamine mesylate salt (Sigma, cat. D9533), restreaked onto fresh GC-Isovitalex-10 µM deferoxamine plates for another overnight growth at 37^o^C with 5% CO_2_. Lawns were resuspended in PBS, OD adjusted to ~0.4 and heat inactivated at 57^o^C for 1 h and then plated to confirm bacterial heat killing. 20 µL/well of bacterial suspension was allowed to dry onto 384 well ELISA plates over 48 h in a biosafety cabinet and plates were stored at 4^o^C until use. Plates were blocked at room temperature for 1 h with 5% bovine serum albumin in PBS (BSA, BioShop Canada, cat. ALB001), and serum was added overnight at 4°C at a dilution of 1:4,000 for cross-reactivity ELISAs or 2-fold serial dilutions for endpoint titres in 1% BSA. After washing, secondary antibody was added at 1:10,000 dilution (Jackson ImmunoResearch, cat. 115-035-003) and subsequently developed with KPL SureBlue TMB Microwell Peroxidase Substrate (SeraCare, cat. 5120-0077). Endpoint titre was calculated as the highest dilution to give a signal over background + 3 standard deviations.
