# Supplementary material for: Rationally designed minimized TbpB confers broad protection against meningococcal infection
Source: PLoS Pathog. 2026 Aug 10;22(8):e1014493. doi: 10.1371/journal.ppat.1014493 (PMC13480600; doi:10.1371/journal.ppat.1014493)
Supplement: Peer review history

# Author comment: Author response to Decision Letter 0

**DOI:** 10.1371/journal.ppat.1014493.r001

28 Jan 2026

Attachment Submitted filename: Response to Editors.pdf

# Peer review: Decision Letter 0

**DOI:** 10.1371/journal.ppat.1014493.r002

**Authors:** Eva Heinz, Vladimir Pelicic

17 Mar 2026

Rationally designed minimized TbpB confers broad protection against meningococcal infection

PLOS Pathogens

Dear Dr. Gray-Owen,

Thank you for submitting your manuscript to PLOS Pathogens. After careful consideration, we feel that it has merit but does not fully meet PLOS Pathogens's publication criteria as it currently stands. Therefore, we invite you to submit a revised version of the manuscript that addresses the points raised during the review process.

Please submit your revised manuscript by May 16 2026 11:59PM. If you will need more time than this to complete your revisions, please reply to this message or contact the journal office at plospathogens@plos.org. When you're ready to submit your revision, log on to https://www.editorialmanager.com/ppathogens/ and select the 'Submissions Needing Revision' folder to locate your manuscript file.

Please include the following items when submitting your revised manuscript:

* A letter that responds to each point raised by the editor and reviewer(s). You should upload this letter as a separate file labeled 'Response to Reviewers'. This file does not need to include responses to any formatting updates and technical items listed in the 'Journal Requirements' section below.

* A marked-up copy of your manuscript that highlights changes made to the original version. You should upload this as a separate file labeled 'Revised Manuscript with Track Changes'.

* An unmarked version of your revised paper without tracked changes. You should upload this as a separate file labeled 'Manuscript'.

If you would like to make changes to your financial disclosure, competing interests statement, or data availability statement, please make these updates within the submission form at the time of resubmission. Guidelines for resubmitting your figure files are available below the reviewer comments at the end of this letter.

We look forward to receiving your revised manuscript.

Kind regards,

Vladimir Pelicic

Academic Editor

PLOS Pathogens

Eva Heinz

Section Editor

PLOS Pathogens

Editor-in-Chief

PLOS Pathogens

Editor-in-Chief

PLOS Pathogens

orcid.org/0000-0002-7699-2064

**Additional Editor Comments:**

The Reviewers, who are experts in the field, felt that the finding that structure-informed engineered loopless C-lobe (LCL) variant of meningococcal Tbp elicits bactericidal antibodies with improved cross-protective potential against both homologous and heterologous strains of N. meningitidis is important. The Reviewers also appreciated 1) the structural characterisation of the LCL antigen, 2) the use of several mouse models for meningococcal infection, and 3) that the manuscript is well written. However, they raise a number of major issues that would need to be addressed, in some occasions by performing additional experiments. In particular, 1) considering the small number of mice/group from which conclusions are drawn and the frequent lack of statistical significance between the groups, many conclusions and titles need to be toned down or should be backed up by performing additional mouse experiments, 2) on a similar note, unlike what is stated in the text, Fig 5E shows that more than 40% of the mice immunised with C-lobe have developed bactericidal responses, hence the conclusion would be different if more mice were included in this particular group, 3) given the previous publications on the use of CL as a backbone for displaying epitopes from other antigens for immunisation, the novelty of the LCL scaffold data reported here should be better explained at the end of the Introduction, and 4) since SBA are important for evaluating any meningococcal vaccine, SBA titres should be determined against heterologous strains to strengthen the central claim that anti-LCL antibodies have wide cross-protective potential.

**Journal Requirements:**

1) We ask that a manuscript source file is provided at Revision. Please upload your manuscript file as a .doc, .docx, .rtf or .tex. If you are providing a .tex file, please upload it under the item type u2018LaTeX Source Fileu2019 and leave your .pdf version as the item type u2018Manuscriptu2019.

2) Please upload all main figures as separate Figure files in .tif or .eps format. For more information about how to convert and format your figure files please see our guidelines:

https://journals.plos.org/plospathogens/s/figures

3) We have noticed that you have uploaded Supporting Information files, but you have not included a list of legends. Please add a full list of legends for your Supporting Information files after the references list.

4) We are unable to open the following Supporting Information file: final_nme_ngo_tbpBs_common.faa. Please kindly revise as necessary and re-upload.

5) Please amend your detailed Financial Disclosure statement. This is published with the article. It must therefore be completed in full sentences and contain the exact wording you wish to be published.

State what role the funders took in the study. If the funders had no role in your study, please state: "The funders had no role in study design, data collection and analysis, decision to publish, or preparation of the manuscript.".

If you did not receive any funding for this study, please simply state: u201cThe authors received no specific funding for this work.u201d

**Reviewers' Comments:**

Reviewer's Responses to Questions

**Part I - Summary**

Please use this section to discuss strengths/weaknesses of study, novelty/significance, general execution and scholarship.

Reviewer #1: This is an interesting paper that advances the long history of Tbps as vaccine antigens. The authors have created a loopless C-lobe of TbpB that has improved vaccine properties to previous constructs. Tbps have application beyond N. meningitidis to other pathogens and so there will be considerable interest in this paper. Th team have used appropriate methods for proof of concept experiments and there will require more exhaustive studies to confirm the vaccine potential but these are beyond the scope of this paper. The manuscript is clearly written and includes appropriate background and discussion.

Reviewer #2: The authors have designed a 'pared back' version of a Neisseria lipoprotein, TbpB, as a vaccine. The rationale, outlined by the authors in the Introduction, is to remove variable and structurally flexible regions of the molecule, to generate LCL. LCL lacks the N lobe of TbpB, as well as sections of the C lobe. They have then undertaken an extensive series of relevant experiments to evaluate the structure/stability and vaccine candidacy of LCL, compared with full length TbpB and the intact C lobe of TbpB only.

The results are interesting as LCL exhibits similar immunogenicity and protection as full length TbpB against the homologous protein or strain expressing the homologous protein, with more evidence of cross reactivity against heterologous proteins, providing support for their rationale.

The paper is well written, and the results/figures are generally laid out clearly but can be improved. There is some overreach in the conclusions they draw from their results.

Reviewer #3: The manuscript by Islam and colleagues reports on the vaccine potential of a “loopless C-lobe” TbpB protein from Neisseria meningitidis. Previously this group had engineered this protein, abbreviated LCL, as a backbone for displaying loops from other antigens for immunogenicity. Here they show the LCL itself is sufficient to elicit bactericidal antibodies against both homologous and heterologous strains of N. meningitidis, based on phylogenetic analysis of TbpB alleles. The N. meningitidis LCL does not cross-protect against genital infection with N. gonorrhoeae and does not elicit antibodies that recognize N. gonorrhoeae or confer bactericidal activity. A strength of the study is the discovery that LCL can serve as an immunogen, with promise as a vaccine antigen since it has greater stability and protease resistance in vitro than the intact C-loop or full-length TbpB. Another strength is the use of two different mouse models for infection by pathogenic Neisseria. However, this is tempered by the lack of novelty of the LCL system, the small number of mice per group from which conclusions are drawn, and the lack of investigation for how LCL elicits protective effects in both systemic and mucosal challenge.

Reviewer #4: This manuscript is of strong interest because it provides a careful and detailed characterization of the immune response induced byan engineered variant of the Tbp C-lobe lacking the most variable loops.The study shows that selective removal of immunodominant and highly variable regions can improve cross-protective potential, possibly by promoting antibody responses toward conserved epitopes. Importantly, structural analyses demonstrate that the overall fold of the C-lobe is preserved after loop deletion, supporting the feasibility of this rational antigen-design strategy.

**********

**Part II – Major Issues: Key Experiments Required for Acceptance**

Please use this section to detail the key new experiments or modifications of existing experiments that should be absolutely required to validate study conclusions.

Generally, there should be no more than 3 such required experiments or major modifications for a "Major Revision" recommendation. If more than 3 experiments are necessary to validate the study conclusions, then you are encouraged to recommend "Reject".

Reviewer #1: The manuscript makes a logical argument for the vaccine potential of the loopless C-lobe of Tbp. The authors acknowledge the limitation of the animal models and immunoassays but the data supports the claims presented.

Reviewer #2: To strengthen the section on the cross-reactivity of anti-LCL antibodies (a central aspect of this work), SBA titers should be determined against heterologous strains. This would enhance the relevance of results and comments about strain coverage (325) even if no SBA titer is detected. SBA results are important for evaluating the candidacy of any meningococcal vaccine.

Reviewer #3: 1. While the trend is promising, mouse experiments are insufficiently powered to make firm conclusions. For example, Figure 3 uses 3-4 mice per group, Figure 4 5-8 mice per group. Power calculations should be provided in Methods.

2. In Figure 5, serum samples from different mouse strain backgrounds and immunization methods should not be grouped together for statistical analysis.

3. Given the variability in response for individual mice, it is worth exploring the immune responses in those mice that were protected compared with those that were not. For instance, what regions of TbpB are targets of the antibodies elicited by LCL? When compared with full-length TbpB or C-lobe, this information would reveal protective antigenic regions of TbpB to refine it as a vaccine antigen. Also, are there differences in the IgG type, or in IgM or IgA response that is elicited? These analyses would greatly increase novelty of the findings.

4. For strains that were heterologously protected by LCL, provide a sequence alignment of their TbpB C-lobe to NmeM982, since the phylogenetic tree in Fig. 6A is for the entire TbpB protein.

5. Experiments examining antibody reactivity in serum of mice immunized with different TbpB-based antigens need to show that TbpB is induced in the strains tested. A M982 delta TbpB control needs to be included as well, since the parents of the two delta TbpB strains used in Fig. S2, FA19 and B1686, do not react with the immunized serum.

Reviewer #4: The rationale for the choice of the allele expressed and the challenge strain should be clearly stated in the first paragraphs.

Figure S1A contains important information and would be more appropriately placed in the main text. In addition, the rationale for the use of different adjuvants should be clarified, including whether differences in the immune response were expected or observed. The reduced protection observed when the two lobes were used in combination also deserves further explanation.

The number of mice used in the two challenge experiments (Figure 3 and S1) differs. Was variability observed in previous experiments considered when selecting only four mice for the experiment shown in Figure 3?

While the use of different mice genotypes to assess immunogenicity and protection is understandable, the rationale for selecting these specific mouse models for the different immunization routes is not explained. In particular, clarification is needed to why both hCEACAM1+/− (mCEACAM1+/+) FvB mice and hCEACAM1+/− (mCEACAM1−/−) C57BL/6 mice were included, and what specific biological question this comparison was designed to address .

Line 251: The rationale for using pre-immune and post-immune sera derived from different experiments should also be clarified.

Line 268: The authors state that the C-lobe induces no bactericidal activity; however, some mice appear to have developed bactericidal responses. This statement should therefore be reconsidered or better contextualized. The authors also mention a correlation between bactericidal activity and protection from sepsis. It would be important to know whether similar evidence was observed in the experiments presented here.

The evidence of cross-recognition is convincing and clearly presented. However, does ELISA reactivity correlate with functional cross-bactericidal activity?

The experiment shown in Figure 7 clearly indicates cross-protection following LCL immunization. It would be informative to report protection outcomes in terms of survival and bacterial clearance kinetics post-infection. In addition, the rationale for the bacterial dose selected for strains carrying different alleles should be provided.

Finally, the protection data reported in Figure S3 would strengthen the manuscript if moved to the main text. The results of the challenge experiments should be presented in a consistent format to enable direct comparison across studies.

**********

**Part III – Minor Issues: Editorial and Data Presentation Modifications**

Please use this section for editorial suggestions as well as relatively minor modifications of existing data that would enhance clarity.

Reviewer #1: Line 182 - I suggest using "efficacy" to describe results of a human clinical trial. Here the authors describe protection against IP challenge in mice.

Line 189 - The loss of protection...

Line 216-7 - This sentence is not clear

Line 257 - "higher IgG titres in ..." Panels A and B do not show titres, rather they show OD at a fixed dilution of serum.

Line 266 and following - "significant bactericidal activity...." The bactericidal activity is generally low and many animals do not respond with any bactericidal activity. The authors should make this clear. I would have like to have seen cross-strain SBA reported.

Line 304 - Are the protein ELISAs Fig 6C whole cell ELISAs or isolated recombinant Tbps?

Fig. 6D - It is a pity this is a single dilution ELISA. Were doubling dilution ELISA performed. All thecross-strain ODs seen low and it's hard to tell if they are really much different to background. It is also a pity that strain M982 was not included as a "negative control"

Line 458 - efficacy = protection

Line 543 - it would be helpful in include a brief description of the SBA method as this is important for this study.

Reviewer #2: The authors should give the serogroup of strains when they are first mentioned in the text.

The sequences deleted to generate LCL are not obvious from the structures shown in Figure 1; only one view of each protein is presented and the deleted regions are mostly unstructured so difficult to see. The authors should include an alignment of the amino acid sequence of LCL with the intact C lobe (ie the sequence of proteins shown in Panels B and C). Some quantification of conserved/variable in the color code should be provided in the figure and/or text.

Figure 2: the authors should provide the original images of proteolytic degradation in the supplementary information. L176 delete enzyme.

Text associated with Figures 4, 7 and 8, and conclusions in the Discussion: the authors need to be careful when describing their results given the lack of statistical significance between the groups in many instances. The titles of the associated sections and the conclusions need to be changed as often only trends towards protection (and not significant protection) were observed.

Figure 4: what was the bacterial burden on other days? In previous work with this model, the group has assessed carriage on day 1 not day 3.

251/252: the authors quote the wrong figures. Same errors in the legend of Figure 5.

Figure 5: results include data from two different mouse genotypes: these should be split into separate columns for clarity.

Figure 5E: Using terminal sera from mice that had been colonized (rather than pre-challenge) might result in boosting of anti-TbpB responses through carriage; this would not be seen in mice immunized with Alum alone. Therefore, part of the SBA results will reflect carriage as well as immunization. The authors should include this caveat in the Results/Discussion.

SBA titers were determined with rabbit complement which can overestimate titers compared with human complement which has become the standard.

Reviewer #3: 1. Line 61: there is not sufficient analysis in this manuscript to state that the LCL “focuses the immune response”. As written, the sentence on lines 58-62 implies there is cross-protection against N. gonorrhoeae, which needs to be clarified.

2. Mouse clinical scoring system needs to be described in Materials and Methods.

3. Reword subjective terms like “robust levels” (line 248) and “highly cross-reactive” (line 298).

4. Raw data in Figure 6B should be provided in a supplemental table.

5. Please explain why some experiments use alum and others Emulsigen-D as adjuvant.

Reviewer #4: (No Response)

**********

PLOS authors have the option to publish the peer review history of their article (what does this mean?). If published, this will include your full peer review and any attached files.

If you choose “no”, your identity will remain anonymous but your review may still be made public.

**Do you want your identity to be public for this peer review?**  For information about this choice, including consent withdrawal, please see our Privacy Policy.

Reviewer #1: **Yes:**  Andrew Gorringe

Reviewer #2: No

Reviewer #3: No

Reviewer #4: **Yes:**  Mariagrazia Pizza

[NOTE: If reviewer comments were submitted as an attachment file, they will be attached to this email and accessible via the submission site. Please log into your account, locate the manuscript record, and check for the action link "View Attachments". If this link does not appear, there are no attachment files.]

**Figure resubmission:**

**Reproducibility:**

?>

# Author comment: Author response to Decision Letter 1

**DOI:** 10.1371/journal.ppat.1014493.r003

10 Jun 2026

Attachment Submitted filename: Response to reviewers.pdf

# Peer review: Decision Letter 1

**DOI:** 10.1371/journal.ppat.1014493.r004

**Authors:** Matthew C. Wolfgang, Vladimir Pelicic

21 Jul 2026

Dear Professor Gray-Owen,

We are pleased to inform you that your manuscript 'Rationally designed minimized TbpB confers broad protection against meningococcal infection' has been provisionally accepted for publication in PLOS Pathogens.

Before your manuscript can be formally accepted you will need to complete some formatting changes, which you will receive in a follow up email. A member of our team will be in touch with a set of requests.

Please note that your manuscript will not be scheduled for publication until you have made the required changes, so a swift response is appreciated.

IMPORTANT: The editorial review process is now complete. PLOS will only permit corrections to spelling, formatting or significant scientific errors from this point onwards. Requests for major changes, or any which affect the scientific understanding of your work, will cause delays to the publication date of your manuscript.

Should you, your institution's press office or the journal office choose to press release your paper, you will automatically be opted out of early publication. We ask that you notify us now if you or your institution is planning to press release the article. All press must be co-ordinated with PLOS.

Thank you again for supporting Open Access publishing; we are looking forward to publishing your work in PLOS Pathogens.

Best regards,

Vladimir Pelicic

Academic Editor

PLOS Pathogens

Eva Heinz

Section Editor

PLOS Pathogens

Sumita Bhaduri-McIntosh

Editor-in-Chief

PLOS Pathogens

orcid.org/0000-0003-2946-9497

Michael Malim

Editor-in-Chief

PLOS Pathogens

orcid.org/0000-0002-7699-2064

***********************************************************

The reviewers are unanimous that their comments have been addressed adequately, which has greatly improved the paper.

Reviewer Comments (if any, and for reference):

Reviewer's Responses to Questions

**Part I - Summary**

Please use this section to discuss strengths/weaknesses of study, novelty/significance, general execution and scholarship.

Reviewer #1: The authors had made a comprehensive response to the reviewer comments. They have addressed mine and other comments and have greatly improved the paper. The manuscript is now suitable for publication.

Reviewer #2: (No Response)

Reviewer #3: The authors have addressed the comments raised in prior review.

Reviewer #4: (No Response)

**********

**Part II – Major Issues: Key Experiments Required for Acceptance**

Please use this section to detail the key new experiments or modifications of existing experiments that should be absolutely required to validate study conclusions.

Generally, there should be no more than 3 such required experiments or major modifications for a "Major Revision" recommendation. If more than 3 experiments are necessary to validate the study conclusions, then you are encouraged to recommend "Reject".

Reviewer #1: None

Reviewer #2: (No Response)

Reviewer #3: (No Response)

Reviewer #4: (No Response)

**********

**Part III – Minor Issues: Editorial and Data Presentation Modifications**

Please use this section for editorial suggestions as well as relatively minor modifications of existing data that would enhance clarity.

Reviewer #1: None

Reviewer #2: (No Response)

Reviewer #3: On the title page, affiliation #4 is used twice. Calgary should be #5.

Reviewer #4: (No Response)

**********

PLOS authors have the option to publish the peer review history of their article (what does this mean?). If published, this will include your full peer review and any attached files.

If you choose “no”, your identity will remain anonymous but your review may still be made public.

**Do you want your identity to be public for this peer review?**  For information about this choice, including consent withdrawal, please see our Privacy Policy.

Reviewer #1: **Yes:**  Andrew Gorringe

Reviewer #2: No

Reviewer #3: No

Reviewer #4: **Yes:**  Mariagrazia Pizza

# Editor report: Acceptance letter

**DOI:** 10.1371/journal.ppat.1014493.r005

**Authors:** Matthew C. Wolfgang, Vladimir Pelicic

Dear Dr. Gray-Owen,

We are delighted to inform you that your manuscript, "Rationally designed minimized TbpB confers broad protection against meningococcal infection," has been formally accepted for publication in PLOS Pathogens.

We have now passed your article onto the PLOS Production Department who will complete the rest of the pre-publication process. All authors will receive a confirmation email upon publication.

The corresponding author will soon be receiving a typeset proof for review, to ensure errors have not been introduced during production. Please review the PDF proof of your manuscript carefully, as this is the last chance to correct any scientific or type-setting errors. Please note that major changes, or those which affect the scientific understanding of the work, will likely cause delays to the publication date of your manuscript. Note: Proofs for Front Matter articles (Pearls, Reviews, Opinions, etc...) are generated on a different schedule and may not be made available as quickly.

Soon after your final files are uploaded, the early version of your manuscript, if you opted to have an early version of your article, will be published online. The date of the early version will be your article's publication date. The final article will be published to the same URL, and all versions of the paper will be accessible to readers.

For Research Articles, you will receive an invoice from PLOS for your publication fee after your manuscript has reached the completed accept phase. If you receive an email requesting payment before acceptance or for any other service, this may be a phishing scheme. Learn how to identify phishing emails and protect your accounts at https://explore.plos.org/phishing.

Thank you again for supporting open-access publishing; we are looking forward to publishing your work in PLOS Pathogens.

Best regards,

Sumita Bhaduri-McIntosh

Editor-in-Chief

PLOS Pathogens

orcid.org/0000-0003-2946-9497

Michael Malim

Editor-in-Chief

PLOS Pathogens

orcid.org/0000-0002-7699-2064
